# Tissue‐specific recombination‐activated clonal and inducible transcription (TRAC‐IT) enables controlled gene activation in stably marked tissues

**DOI:** 10.1111/tpj.71151

**Published:** 2026-09-28

**Authors:** Tristan Wijsman, Jana Wittmer, Robert Winter, Teun Bruins, Arjan van Zeijl, Harm Nijveen, Ben Scheres, Renze Heidstra

**Affiliations:** ^1^ Cell‐and Developmental Biology, Cluster Plant Developmental Biology Wageningen University & Research Droevendaalsesteeg 1 6708 PB Wageningen the Netherlands; ^2^ Rijk Zwaan Breeding B.V. Burgemeester Crezéelaan 40, PO Box 40 2678 ZG De Lier the Netherlands; ^3^ Bioinformatics group Wageningen University & Research Droevendaalsesteeg 1 6708 PB Wageningen the Netherlands

**Keywords:** technical advance, tissue‐specific gene expression, CRE/loxP recombination, inducible transgene activation, Arabidopsis, regeneration

## Abstract

Chemically inducible gene expression systems provide temporal control of transgene activation and therefore represent powerful and widely used tools in plant research. When driven by a cell‐type‐specific promoter, these systems additionally offer spatial control. However, continued transgene activity is necessarily linked to the preservation of that particular cell type, which might be lost as cells (de)differentiate. To overcome this, we present Tissue‐specific Recombination‐Activated Clonal and Inducible Transcription (TRAC‐IT), a two‐step system that uncouples inducible gene expression from cell identity. In the first step, a tissue‐specific expressed CRE‐GR recombinase is activated upon dexamethasone application, triggering loxP recombination and thereby generating tissue‐specific clones of cells expressing *YFP* and *XVE*. YFP marks the clone and the XVE *trans*‐activator allows inducible expression of downstream genes within the clone. In the second step, a subsequent β‐estradiol application activates XVE to induce transgene expression exclusively within these YFP‐marked clones. Because the inducibility of the transgenes becomes genomically anchored, it is maintained upon cell division and/or identity change. TRAC‐IT is compatible with Golden Gate cloning, providing simple variation of tissue‐specific promoters and transgenes. We show that TRAC‐IT supports inducible and controlled tissue‐specific clone formation, and that transgenes can be specifically induced within these clones. We employed TRAC‐IT to demonstrate that epidermal cells in the root meristem can be persuaded to regenerate somatic embryos through clone‐based induction of *PLT1* and *WOX5*. Thus, TRAC‐IT offers a versatile tool that provides new technical possibilities to explore the mechanisms driving plant development in a stable tissue‐specific fashion.

## Significance Statement

Tissue‐specific Recombination‐Activated Clonal and Inducible Transcription (TRAC‐IT), a two‐step system that uncouples inducible gene expression from cell identity. TRAC‐IT offers a versatile tool that provides new technical possibilities to explore the mechanisms driving plant development in a stable tissue‐specific fashion.

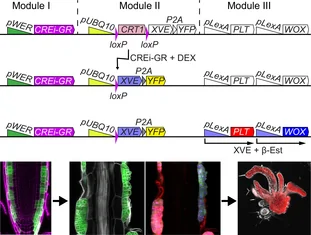

In this work, we introduce TRAC‐IT, a system that enables β‐estradiol‐controlled transgene expression in tissue‐specific clones created by dexamethasone‐triggered CRE/*loxP* recombination. Because transgene inducibility is genomically anchored within clones, it is maintained upon cell division and identity changes, allowing for new experimental opportunities exemplified by the interrogation of tissue‐specific regeneration competence toward *PLT1*/*WOX5* induction.

## INTRODUCTION

Technologies that offer inducible control over transgene expression represent powerful tools in plant research and biotechnology (Moore et al., 2006). In contrast to classical transgene overexpression, where the transgene is simply expressed under control of a constitutive promoter, inducible transactivation systems allow for temporal control of transgene expression as they depend on the presence of an inducing cue. Such a setup is favorable when (1) ectopic expression of transgenes unintentionally hampers plant development, (2) the aim is to study transgene effects only in a particular developmental stage, (3) downstream analysis is desired after a specified time of gene induction.

Over the last three decades, a wide range of inducible systems have been developed, each exploiting the wide variety of gene regulatory mechanisms found in nature. For example, promoters derived from heat shock‐responsive genes such as Arabidopsis *HSP18.2* can be used to activate transcription in response to heat treatment (Freeman et al., 2011; Shinmyo et al., 1998), and the *AlcR/AlcA* transactivation system derived from *Aspergillum nidulans* can be used to activate gene expression following ethanol application (Caddick et al., 1998; Roslan et al., 2001). Other elegant examples are based on animal steroid receptors, of which the β‐Estradiol inducible XVE/*LexA* module (Zuo et al., 2000) and the dexamethasone‐inducible LhGR/*Op6* and GVG/*UAS* modules (Craft et al., 2005) (Aoyama & Chua, 1997) are the most prominent. These systems offer the advantage that the inducing compounds are (synthetic) animal steroid hormones, to which plants are in principle insensitive.

Steroid‐inducible transactivation systems make use of a synthetic transcription factor (STF) and accompanying synthetic target promoter (Moore et al., 2006). The STF typically consists of three domains: a well‐characterized DNA‐binding domain, a transcriptional activation domain, and a ligand‐binding steroid receptor domain. In the case of the XVE/*LexA* module, the XVE STF consists of the DNA‐binding domain of the bacterial LexA repressor protein, the Herpes simplex virus VP16 transcription activation domain, and the carboxyl region of the human ESR1 receptor (Zuo et al., 2000). The synthetic promoter generally contains several repeats of the recognition sequence of the STF's DNA‐binding domain. In the case of the *LexA* promoter, eight copies of the *LexA* operator sequence reside upstream of a minimal *35S* promoter element (Moore et al., 2006; Zuo et al., 2000). When the ligand is bound, the steroid receptor domain undergoes a conformational change (Lockett et al., 2024; Yaşar et al., 2017), thereby activating the STF to drive transcription from the synthetic promoter. Importantly, the concentration of the applied inducer allows for tuning of transgene expression levels by the XVE/*LexA*, LhGR/*Op6* and GVG/*UAS* modules (Aoyama & Chua, 1997; Craft et al., 2005; Zuo et al., 2000). Compared with a direct fusion of the steroid receptor domain to a nuclear or transcription factor of interest (Shimada et al., 2022), these transactivation systems can activate multiple genes using the same synthetic promoter.

The power of the steroid‐inducible transactivation systems is exemplified by a recent study that employed the XVE/*LexA* system to study the role of *MYB74* in the transdifferentiation of non‐epidermal residuum cells into cuticle‐forming epidermal cells after abscission. Induced expression of *MYB74* also enabled profiling of the transcriptional role of MYB74 by allowing a 10‐h induction window (Wen et al., 2025). Multiple inducible systems can also be combined, as shown in a recent study where XVE/*LexA* controlled dedifferentiation genes and GVG/*UAS* controlled stem cell‐related transcription factors revealed that regeneration via somatic embryogenesis can be efficiently induced (Wittmer et al., 2025).

Gene induction can be performed in a tissue‐specific manner by expressing the *STF* under a tissue‐specific promoter (Schürholz et al., 2018; Siligato et al., 2016). For instance, to dissect the role of auxin signaling in vascular tissues during graft formation, a recent study employed a tissue‐specific LhGR/*pOp*‐based system to drive expression of the dominant negative *bdl‐2* allele (Serivichyaswat et al., 2024). Repression of auxin signaling under the procambium‐specific *PXY* and *ATHB8* promoters inhibited tissue attachment, wound‐induced callus formation, and reconnection of phloem and xylem. In another study, XVE/*LexA*‐inducible expression of the *PLT2* transcription factor in the epidermis inhibited root hair formation but not protoxylem differentiation, while expression in the protoxylem had the opposite effect, showing that PLT genes inhibit differentiation locally (Mähönen et al., 2014).

Although tissue‐specific gene induction can be a valuable tool, it suffers from an important technical drawback: the continued tissue‐specific transcription of the STF and thereby its target gene induction is dependent on maintaining tissue identity. For example, inducible expression of transgenes in early lateral root primordia has been successfully performed using the *GATA23* promoter (van Gelderen et al., 2025). However, as the *GATA23* promoter expression is lost from stage V onward (de Rybel et al., 2010), the transgenes will no longer be expressed in later‐stage primordia or developing lateral roots. Furthermore, the induced genes themselves can cause changes in the identity of the cells in which they are expressed, for example, in the case of transcription factor induced regeneration (Horstman et al., 2017; Tang et al., 2025; Zuo et al., 2002).

An approach to overcome the loss of expression due to tissue fate change would be to target the genomic DNA of the specific cell type, thereby generating a so‐called clone of cells with constitutive target gene expression. This principle is utilized in lineage‐dependent knockout systems (Ablain et al., 2015; Decaestecker et al., 2019), where the expression of CRISPR‐Cas9 is controlled by a tissue‐specific promoter. Mutagenesis is restricted to cells in the domain of promoter activity, but the induced mutations are inherited by all daughters of these cells, generating a clone. Similarly, tissue‐specific clones with activated gene expression can be generated with the CRE/*loxP* site‐specific recombination system derived from bacteriophage P1 (Heidstra et al., 2004; Sauer & Henderson, 1988; Wang et al., 2025). Cre activity recombines two direct repeat 34‐nt *loxP* sites, circularizing and effectively deleting the interspacing DNA and generating a clone of recombined cells. Deletion of a *loxP*‐flanked stuffer gene can thereby place a target gene under control of a constitutive promoter, resulting in gene activation that is maintained in all clonal daughter cells, irrespective of their identity (Heidstra et al., 2004; Wachsman & Heidstra, 2010). Although the activity of the CRE itself is not controllable, fusing it to the glucocorticoid receptor domain (GR) makes its activity dexamethasone‐inducible (Brocard et al., 1998; Wachsman et al., 2011).

Here, we integrate the CRE‐GR and XVE/*LexA* modules into a single vector to allow for tissue‐specific recombination‐activated clonal and inducible transcription (TRAC‐IT). We show that a tissue specifically expressed CRE‐GR can form clones in an inducible and tissue‐specific fashion, and that the extent of clone formation can be controlled. Using fluorescent markers, we further demonstrate β‐estradiol‐inducible expression of transgenes specifically within clones. Finally, we apply TRAC‐IT to demonstrate that epidermis cells can regenerate somatic embryos *in situ* by inducible transactivation of *PLT1*/*WOX5*.

## RESULTS

### TRAC‐IT vector design and function

The TRAC‐IT system consists of three modules contained in a single vector (Figure 1a). Module I expresses *Cre‐GR* under the desired cell‐type‐specific promoter, allowing dexamethasone (DEX)‐inducible activation of recombination. Module II functions as the switch for Cre/*loxP*‐mediated recombination. It holds the constitutive *AtUBQ10* promoter that drives the expression of a *loxP*‐flanked stuffer cassette comprising the *CRT1* norflurazon resistance gene and a *Nos* terminator (Heidstra et al., 2004). Downstream of the *loxP*‐enclosed stuffer cassette is an *XVE‐P2A‐erYFP* fusion gene, which is initially not expressed as transcription stops at the Nos terminator in the stuffer. Finally, Module III contains the transgenes of interest under control of XVE‐targeted *LexA* promoters, here exemplified by endoplasmic reticulum (er)‐localized *mCherry* and *erCFP* fluorescent protein genes.

**Figure 1:**
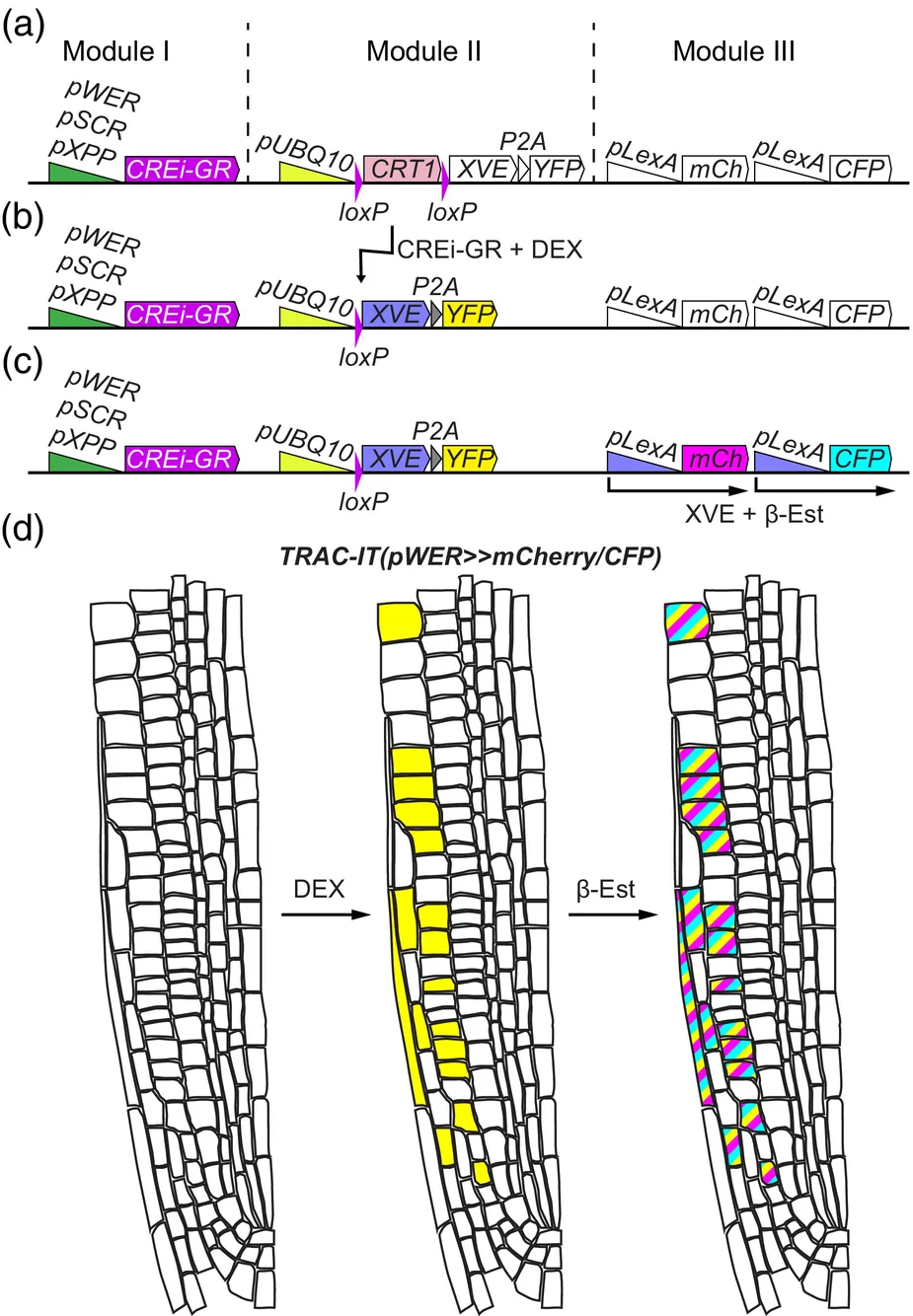
Schematic overview of the TRAC‐IT system. (a) TRAC‐IT clonal induction system exemplified with fluorescent marker genes. Module I harbors *CREi‐GR*, encoding the DEX‐activatable site‐specific recombinase expressed from a tissue‐specific promoter (in this example, *pWER*, *pSCR*, or *pXPP*). In Module II, the *pUBQ10* constitutive promoter drives the expression of a *loxP*‐flanked norflurazon resistance gene (*CRT1*), thereby preventing transcription of the downstream *XVE‐P2A‐erYFP* fusion gene. Module III harbors the *LexA* controlled transgenes (here *ermCherry* [*mCh*] and *erCFP* [*CFP*], respectively). (b) DEX application activates the tissue‐specific expressed CREi‐GR that recombines the *loxP* sites thereby eliminating the *CRT1* gene. *XVE‐P2A‐erYFP* now becomes expressed from the *pUBQ10* promoter. (c) Upon β‐Est application, XVE transactivates the expression of the inducible transgenes within the erYFP‐marked clones (arrows). (d) Schematic visualization of *TRAC‐IT(pWER>>mCherry/CFP)* function in the Arabidopsis root meristem. Application of DEX results in the formation of epidermis and lateral root cap‐specific erYFP‐marked clones, indicated in yellow. Subsequent application of β‐Est induces the expression of ermCherry (magenta) and erCFP (cyan) only in clones.

Upon application of DEX, the tissue specifically accumulated CRE‐GR protein (module I) is activated, resulting in recombination of the two *loxP* sites (Module II). This excises the stuffer cassette from the genome of the cell, activating transcription of *XVE‐P2A‐erYFP*, that is now directly downstream of the constitutive *AtUBQ10* promoter (Figure 1b). By making use of the *P2A* peptide, which is reported to cause ribosomal skipping (Liu et al., 2017), the expressed transcript will be translated into the separate XVE and erYFP proteins, allowing transgene induction in the fluorescently marked cells and their progeny, henceforth named “clones.” Application of β‐estradiol (β‐Est) then causes XVE‐mediated induction of the transgenes in Module III, exclusively in erYFP‐marked clones (Figure 1c,d).

Because the clones are genomically anchored, transgene induction in clones can be maintained irrespective of changes in developmental stage or identity. In addition, the cell lineage in the clone expresses erYFP, allowing its detection at any desired experimental stage.

### The P2A peptide allows formation of functional XVE and erYFP proteins

To allow for transgene induction in marked clones using TRAC‐IT, we employed the 22 amino acid long P2A peptide derived from the Porcine teschovirus (Donnelly et al., 2001; Liu et al., 2017) to facilitate the translation of separate XVE and erYFP proteins from a single transcript.

To verify that *P2A* separated *XVE* and *erYFP* are translated as individual proteins, we compared protein accumulation of transiently transformed *p35S::erYFP* and *p35S::XVE‐P2A‐erYFP* constructs in leaves of *Nicotiana benthamiana* (Nicotiana) by means of agroinfiltration. Four days after infiltration, the presence of erYFP fluorescence in leaf tissue was confirmed for both constructs using a confocal microscope (Figure S1a,b). Next, we performed a Western blot with anti‐GFP antibodies on triplicate protein samples of each construct. In all protein samples of *p35S::erYFP* and *p35S::XVE‐P2A‐erYFP* infiltrated leaves, we detected a strong band at ~27 kDa, indicative of erYFP, while a band of ~85 kDa, indicative of non‐cleaved XVE‐P2A‐erYFP protein, was not detected in any sample (Figure S1c). This shows that *XVE‐P2A‐erYFP* mRNAs are effectively translated into separate XVE and erYFP proteins in Nicotiana.

After translation, 21 amino acids of the P2A peptide will remain attached to the C‐terminus of XVE. To ensure that this does not inhibit the activity of XVE, we co‐agroinfiltrated Nicotiana leaves with constructs *p35S::XVE‐P2A‐erYFP* and *pLexA::erCFP*. As a positive control, we co‐agroinfiltrated *p35S::XVE* (lacking the additional 21 aa) and *pLexA::erCFP* constructs. To examine XVE‐induced *erCFP* expression, the leaves were infiltrated with 10 μM β‐Est or a mock solution 3 days after the initial construct infiltration. The next day, we performed confocal microscopy analysis and observed that erCFP fluorescence was strongly and specifically enhanced by infiltration of β‐Est for both construct combinations (Figure S1d–g). This shows that the XVE protein resulting from cleavage of XVE‐P2A‐erYFP can be effectively used to induce gene expression.

### Applying TRAC‐IT to induce tissue‐specific clones

To enable the use of the TRAC‐IT system in Arabidopsis roots, we first tested marker lines for a selection of reported promoters for tissue specificity along the developmental zones of the primary root. We employed the *WEREWOLF* promoter (*pWER*) for lateral root cap (LRC) and epidermis, the *CORTEX2B* (*pCO2B*) for cortex, the *SCARECROW* promoter (*pSCR*) for endodermis, quiescent center, and cortex–endodermis initial and its daughter (QC/CEI(D)), the *SHORT‐ROOT* promoter (*pSHR*) for vascular tissues, the xylem pole pericycle promoter (*pXPP*), and the *WUSHEL‐RELATED HOMEOBOX 5* promoter (*pWOX5*) for QC (Table 1; Figure S2).

**Table 1:** Promoters that were confirmed in this study to exhibit tissue‐specific expression in the meristem and differentiation zone of the Arabidopsis primary root at 5–7 dpg

Promoter	Tissue	Downstream locus	Fragment length (bp)	References
pWER	Epidermis/LRC	AT5G14750	4272	Lee and Schiefelbein (1999)
pCO2B	Cortex	AT1G62500	3324	Heidstra et al. (2004) and Denyer et al. (2019)
pSCR	Endodermis/QC/CEI(D)	AT3G54220	2234	Malamy and Benfey (1997)
pXPP	Xylem pole pericycle	AT4G30450	2499	Andersen et al. (2018)
pSHR	Vasculature	AT4G37650	2442	Nakajima et al. (2001)
pWOX5	QC	AT3G11260	3512	Blilou et al. (2005) and Sarkar et al. (2007)

However, in a first test of the full TRAC‐IT construct, containing the *SCR* promoter, we found that most T1 seedlings (109/123) exhibited constitutive YFP accumulation without DEX induction (Figure S3a), due to CRE/*LoxP* recombination in *Agrobacterium* (Figure S3b). We therefore employed the intronized *CREi‐GR* in all subsequent constructs, which prevents premature recombination (Figure S3c).

With the recombination problems solved, we tested whether the *CREi‐GR*/*LoxP‐CRT1‐LoxP* recombination system used in TRAC‐IT can generate clones in any desired root cell type. To this end, we generated simple reporter‐clone testing constructs driven by the above‐mentioned promoters. These constructs contain the same Module I and Module II cassettes as TRAC‐IT, with the *XVE‐P2A‐erYFP* gene replaced by an *erGFP* reporter (Figure S4a). Following a 1‐day DEX induction, confocal analysis on two independent lines per promoter‐construct showed that clone formation is mostly tissue‐specific for all six tested promoters (Figure S4b). This demonstrates that the *CREi‐GR*/*LoxP‐CRT1‐LoxP* setup used in the TRAC‐IT system is capable of clone formation across all root tissues tested.

To characterize TRAC‐IT driven clone formation, we generated three TRAC‐IT constructs: *TRAC‐IT(pWER>>mCherry/CFP)* for epidermis and LRC, *TRAC‐IT(pSCR>>mCherry/CFP)* for endodermis, QC, and CEI(D), and *TRAC‐IT(pXPP>>mCherry/CFP)* for the xylem pole pericycle (Figure 1; Figure S2). Based on T‐DNA copy number, we selected 10 independent T2 lines for each of the three TRAC‐IT constructs. To comprehensively assess the performance of TRAC‐IT across these 30 lines and guide selection of lines for further analysis, we performed a simple and high‐throughput screening using a fluorescence binocular microscope. Six‐day‐old seedlings were treated with either 10 μM DEX or a mock for 24 h, after which we assessed the presence and quantity of clones. For all lines, we observed a clear increase of clone‐derived YFP fluorescence in DEX‐treated seedlings compared with mock (Table S1; Figure S5; File S1). In line with the activity of the promoter, TRAC‐IT(pXPP>>mCherry/CFP) lines did not show clone formation in the root meristem, but rather in the differentiation zone, starting at 1.5–5 mm from the root tip, depending on the line (Table S1). Lines of TRAC‐IT(pWER>>mCherry/CFP) and TRAC‐IT(pSCR>>mCherry/CFP), on the other hand, did form clones in the root meristem, and induced clones were generally observed up to 5–9 mm above the root tip (Table S1). Western blot analysis on stable TRAC‐IT lines confirmed successful P2A‐mediated cleavage of *XVE‐P2A‐erYFP*, as observed in Nicotiana (Figure S1h).

Occasional clones were observed in mock‐treated roots across all lines, indicating low levels of non‐induced recombination. We quantified this in the binocular screening assay by scoring the proportion of roots containing non‐induced clones in the root meristem for TRAC‐IT(pWER>>mCherry/CFP) and TRAC‐IT(pSCR>>mCherry/CFP) lines, and in a 5‐mm section of the differentiation zone for TRAC‐IT(pXPP>>mCherry/CFP) lines (Table S1). Across transgenic lines, we observed that the percentage of roots exhibiting non‐induced clones showed a positive correlation with T‐DNA copy number (*R* ^2^ = 0.66, Figure S6).

For further analysis, we selected one single‐copy line per promoter‐construct that showed a relatively low number of non‐induced clones. To characterize clone formation with more resolution, we induced 6‐day‐old homozygous T3 seedlings with 10 μM DEX or a mock for 24 h. Following induction, seedlings were transferred back to media without inducer for 8 h and subsequently imaged for YFP‐marked clones with a confocal microscope. We observed that clones in TRAC‐IT(pWER>>mCherry/CFP) line T3‐1 were predominantly observed in the epidermis and LRC (Figure 2a,b), while clones in TRAC‐IT(pSCR>>mCherry/CFP) line T3‐26 were mostly found in the endodermis (Figure 2c,d). This clone formation pattern was found in both the root meristem (Figure 2a,c) and differentiation zone (Figure 2b,d). For TRAC‐IT(pXPP>>mCherry/CFP) line T3‐29, we only observed clones in the differentiation zone, mainly in the xylem pole pericycle (Figure 2e,f).

**Figure 2:**
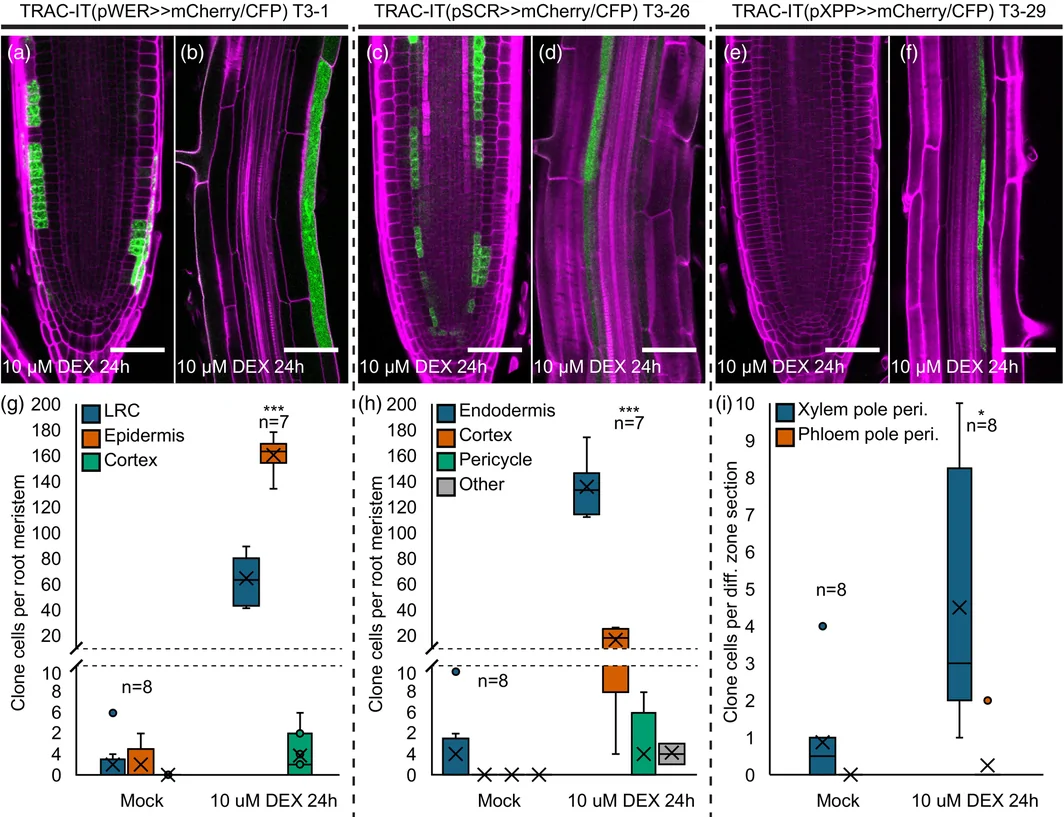
TRAC‐IT clone formation in Arabidopsis. (a–f) Representative confocal images showing clone formation visualized by erYFP in the root of homozygous, single‐copy lines TRAC‐IT(pWER>>mCherry/CFP) T3‐1 (a, b), TRAC‐IT(pSCR>>mCherry/CFP) T3‐26 (c, d), and TRAC‐IT(pXPP>>mCherry/CFP) T3‐29 (e, f). (a, c, e) meristem clones and (b, d, f) differentiation zone clones observed following 24 h induction with 10 μM DEX. YFP fluorescence in false green, Renaissance SR2200 cell wall staining in magenta. (g–i) Quantification of the number of clone cells (YFP‐positive) in the root meristem for the same lines of *TRAC‐IT(pWER>>mCherry/CFP)* (g) and *TRAC‐IT(pSCR>>mCherry/CFP)* (h), and in a root section at ~15 mm from the tip for *TRAC‐IT(pXPP>>mCherry/CFP)* (i), following 24 h induction with mock or 10 μM DEX. Boxplots represent the number of clone cells counted in each tissue from individual root meristem or differentiation zone z‐stacks. *n* indicates the number of roots analyzed. Statistics performed with a two‐tailed student's *t*‐test, assuming unequal variance. *P < 0.05; ***P < 0.001. Scale bars: 50 μm.

We then quantified the DEX‐induced and non‐induced clone cells (File S2) using z‐stacks recorded for root meristems of TRAC‐IT(pWER>>mCherry/CFP) line T3‐1 (Figure 2g) and TRAC‐IT(pSCR>>mCherry/CFP) line T3‐26 (Figure 2h), and differentiation zone sections at ~15 mm from the root tip for TRAC‐IT(pXPP>>mCherry/CFP) line T3‐29 (Figure 2i). From the total of 1585 clone cells counted across 7 DEX‐induced root meristems of TRAC‐IT(pWER>>mCherry/CFP) line T3‐1, 451 (28.5%) and 1121 (70.7%) were respectively found in the LRC and epidermis. All 13 (0.8%) non‐specific clone cells were found in the cortex (Figure 2g). From the total of 1092 DEX‐induced clones (*n* = 7 meristems) counted in TRAC‐IT(pSCR>>mCherry/CFP) line T3‐26, 948 (86.8%) were found in the endodermis (including initials), and 9 (0.8%) were found in the QC. Non‐specific clones were found in the cortex (115, 10.5%), pericycle (14, 1.3%), and vasculature (2, 0.2%) (Figure 2h). For TRAC‐IT(pXPP>>mCherry/CFP) line T2‐29 (*n* = 8 roots), 36/38 (94.7%) DEX‐induced clones were found in the xylem pole pericycle, while the two remaining clones (5.3%) were found in the phloem pole pericycle (Figure 2i). Although present at a lower quantity, all clone cells observed in the mock‐treated roots were found in the tissues targeted by the respective promoter used (Figure 2g–i).

To confirm the tissue specificity of clone formation, we performed further confocal analysis on both DEX (Figure S7) and mock‐treated (Figure S8) T2 seedlings of the 27 other TRAC‐IT lines. We found that, in all lines, both induced and non‐induced clones were predominantly found in the targeted tissues. Combined, our results demonstrate that TRAC‐IT enables inducible, predominantly tissue‐specific clone formation in the background of a minimal number of non‐induced clones that were always observed in the targeted tissue.

Given that the activity of GR in protein fusions is dose‐dependent and reversible with respect to DEX application (Horstman et al., 2017; Nara et al., 2000), we hypothesized that we could tune the number of clones by altering the concentration of DEX or the duration of the induction. Hence, we performed a time‐course experiment with inductions of 2, 8, and 24 h on 10 μM DEX as well as a 24‐h induction with a 0.1, 1, and 10 μM DEX concentration series on two of the previously characterized TRAC‐IT lines: TRAC‐IT(pWER>>mCherry/CFP) line T3‐1 (Figure 3a,b), and TRAC‐IT(pSCR>>mCherry/CFP) line T3‐26 (Figure 3c,d). Following clone quantification (File S2) as described above, we observed a statistically significant increase in the number of clone cells along with increments of induction time (Figure 3a,c) and concentration (Figure 3b,d) in both lines, demonstrating tunable application of TRAC‐IT clone formation.

**Figure 3:**
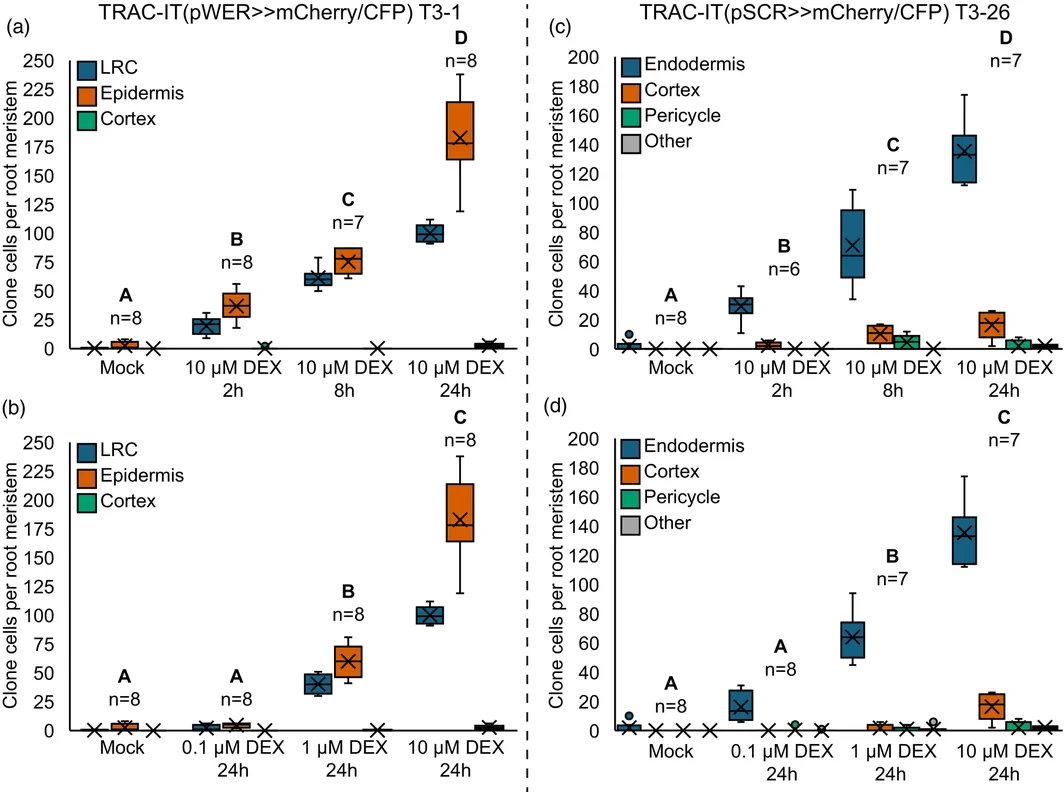
Quantification of scalable clone induction. (a–d) Number of YFP‐marked clone cells in the root meristem of TRAC‐IT(pWER>>mCherry/CFP) T3‐1 (a, b) and TRAC‐IT(pSCR>>mCherry/CFP) T3‐26 (c, d) seedlings, following a 2, 8, or 24 h induction on 10 μM DEX (a, c) or a 24 h induction on 0.1, 1, or 10 μM DEX (b, d). Boxplots represent the number of clone cells counted in each tissue from individual root meristem or differentiation zone z‐stacks. *n* indicates the number of roots analyzed. Statistical groups (*P* < 0.05) were determined with Tukey's HSD test within each experiment and indicated with capital letters in bold.

### TRAC‐IT clone‐specific inducible expression of transgenes

We next tested the TRAC‐IT system for functionality of Module III, in the current setup harboring *ermCherry* and *erCFP* fluorescent marker genes to allow visual analysis of induced activation of transgene expression within clones. Six‐day‐old seedlings from the same 30 T2 lines as described above were sequentially transferred to 10 μM DEX for 24 h to induce clones, followed by 10 μM β‐Est for 24 h to induce expression of the Module III genes *ermCherry* and *erCFP*. Using a fluorescence binocular, we screened for mCherry, CFP, and YFP fluorescence before and after β‐Est induction (Table S1). A total of 25 lines showed the expected mCherry and CFP fluorescence only after induction, which also colocalized with the clonal YFP fluorescence. Three lines showed inducible fluorescence of mCherry but not CFP, one line showed neither mCherry nor CFP fluorescence, and one line exhibited constitutive mCherry fluorescence in the absence of CFP fluorescence regardless of induction. Three appropriately responding T2 lines per TRAC‐IT construct (Table S1) were imaged in more detail with a confocal microscope. In all lines, we observed that ermCherry and erCFP were only present upon induction, and then only in cells that also accumulated erYFP (Figure 4). The fluorescence in the CFP emission range that was observed in the meristem outside of clones, regardless of induction, was identified in wild‐type roots as autofluorescence (Figure S9). This shows that the TRAC‐IT system allows for inducible transgene expression in selectively generated clones.

**Figure 4:**
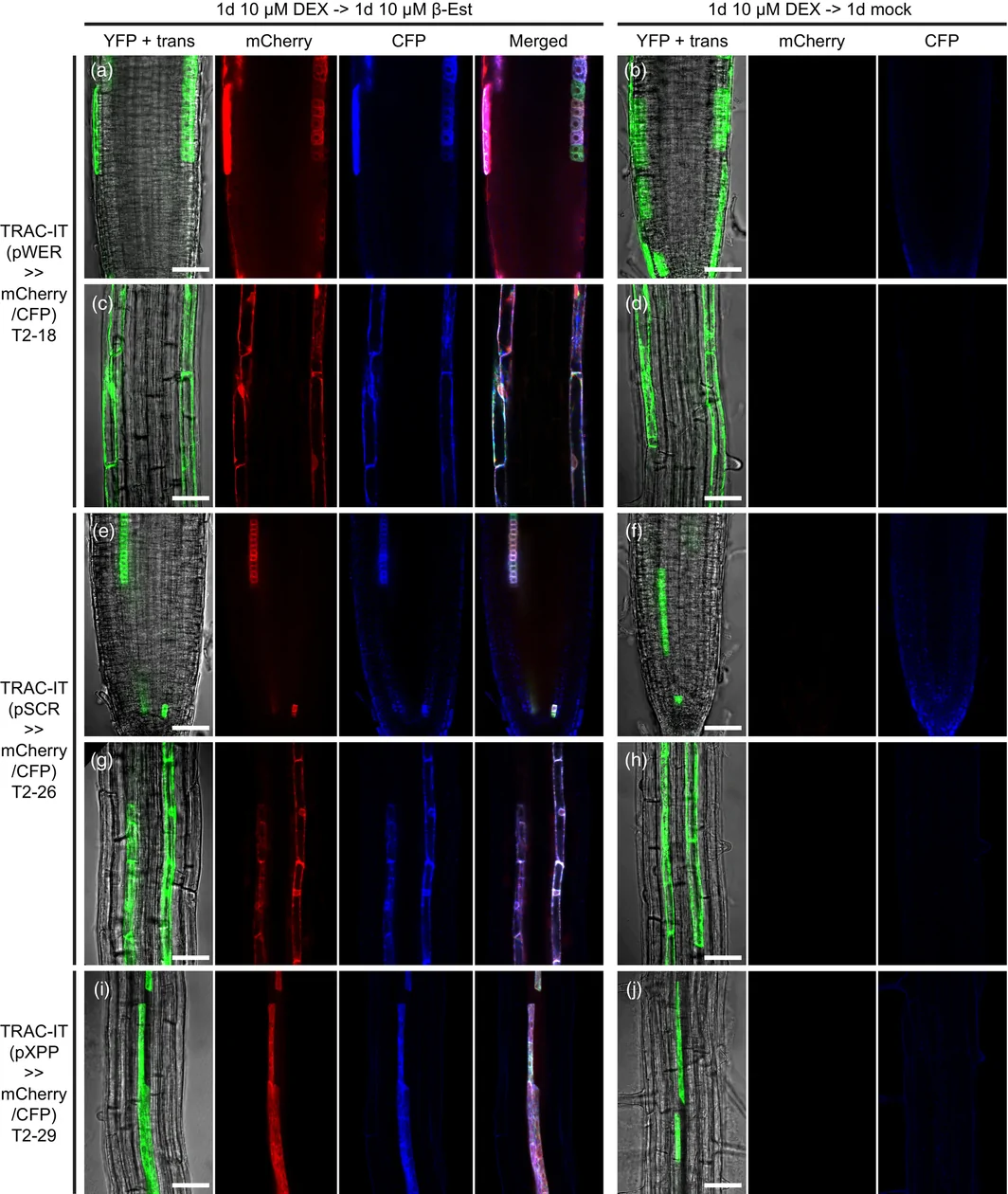
Controlled and clone‐specific induction of *ermCherry* and *erCFP* transgenes using the TRAC‐IT system. (a–j) Representative confocal images of roots from TRAC‐IT(pWER>>mCherry/CFP) line T2‐18 (a–d), TRAC‐IT(pSCR>>mCherry/CFP) line T2‐26 (e–h), and TRAC‐IT(pXPP>>mCherry/CFP) line T2‐29 (i, j). Images show the root meristem (a, b, e, f) or differentiation zone (c, d, g–j). All roots were first treated with 10 μM DEX for 24 h to induce clone formation, followed by a 24 h treatment with 10 μM β‐Est (a, c, e, g, i) or a mock control (b, d, f, h, j). Each confocal image is presented as three separate panels showing YFP fluorescence (green) overlaid with transmitted light (grayscale), mCherry fluorescence (red) and CFP fluorescence (blue). For β‐Est‐treated samples (a, c, e, g, i), an additional fourth panel is included in which the YFP, mCherry, and CFP channels are overlaid using the same color scheme. Scale bars: 50 μm.

Unexpectedly, we observed low levels of mCherry fluorescence in cells adjacent to cloned cells in all lines upon β‐Est induction (Figure S10a). Closer analysis showed that the mCherry fluorescence in non‐clone cells did not localize to the ER, but rather to the nucleus and cytoplasm (Figure S10b), with the signal weakening as the distance from the clone increased (Figure S10c). From these observations, we conclude that, next to er‐localized mCherry, clones also produced free mCherry protein, which is highly cell‐to‐cell mobile (Rim et al., 2011). Possibly, this is caused by the initiation of translation from an alternative start codon within the mCherry sequence (Fages‐Lartaud et al., 2022), or by otherwise incomplete targeting of the translated protein to the ER by the ribosome.

Together, our results show that the TRAC‐IT system is tailor‐made for tunable clone formation and inducible transgene expression in the generated clones.

### Induction of PLT1/WOX5‐induced regeneration from root epidermal cells using TRAC‐IT

We previously showed that somatic embryos regenerate on Arabidopsis roots upon the induced expression of the root stem cell factors *PLT1* and *WOX5* (Wittmer et al., 2025). Confocal analysis on regenerating roots at early timepoints revealed that periclinal divisions in the meristem were mainly observed in the epidermis and cortex, while they were almost exclusive to the pericycle in the differentiation zone. This suggested that meristematic epidermis and cortex cells can undergo regeneration with the PLT/WOX system, contrasting with classical hormone‐mediated regeneration, in which the pericycle gives rise to callus competent for regeneration in both meristematic and differentiated root tissues (Atta et al., 2009; Wittmer et al., 2025).

To test whether cells in the meristematic epidermis can indeed regenerate somatic embryos upon ectopic expression of *PLT1* and *WOX5* only in these cells, we generated lines harboring *TRAC‐IT(pWER>>PLT1/WOX5)*. In this construct, TRAC‐IT allows for transactivation of *PLT1* and *WOX5* in *pWER*‐induced epidermal clones. To prevent cell‐to‐cell mobility of encoded proteins, *PLT1* and *WOX5* were fused to a triple *mCherry* and double *GFP*, respectively (Berckmans et al., 2020; Mähönen et al., 2014). This additionally allowed for visual confirmation of their expression. From a population of 25 T2 lines, nine lines displayed some degree of regeneration after clone induction, a fraction that is comparable to the 5/23 T2 lines regenerating upon constitutive PLT1/WOX5 overexpression (Wittmer et al., 2025). We selected the best performing homozygous line in terms of regeneration (T3‐23) for further analysis. Using this line, we observed clone formation in the epidermis and LRC following 24 h of DEX induction, with occasional clones also forming in the cortex (Figure 5a,b). Next, we induced regeneration from epidermal clones by subsequently transferring seedlings to 10 μM β‐Est media. We marked the position of the root tip because, in contrast to their constitutive induction in all tissues, epidermis clone‐specific induction of *PLT1* and *WOX5* did not stop root growth (Wittmer et al., 2025) (Figure 5c).

**Figure 5:**
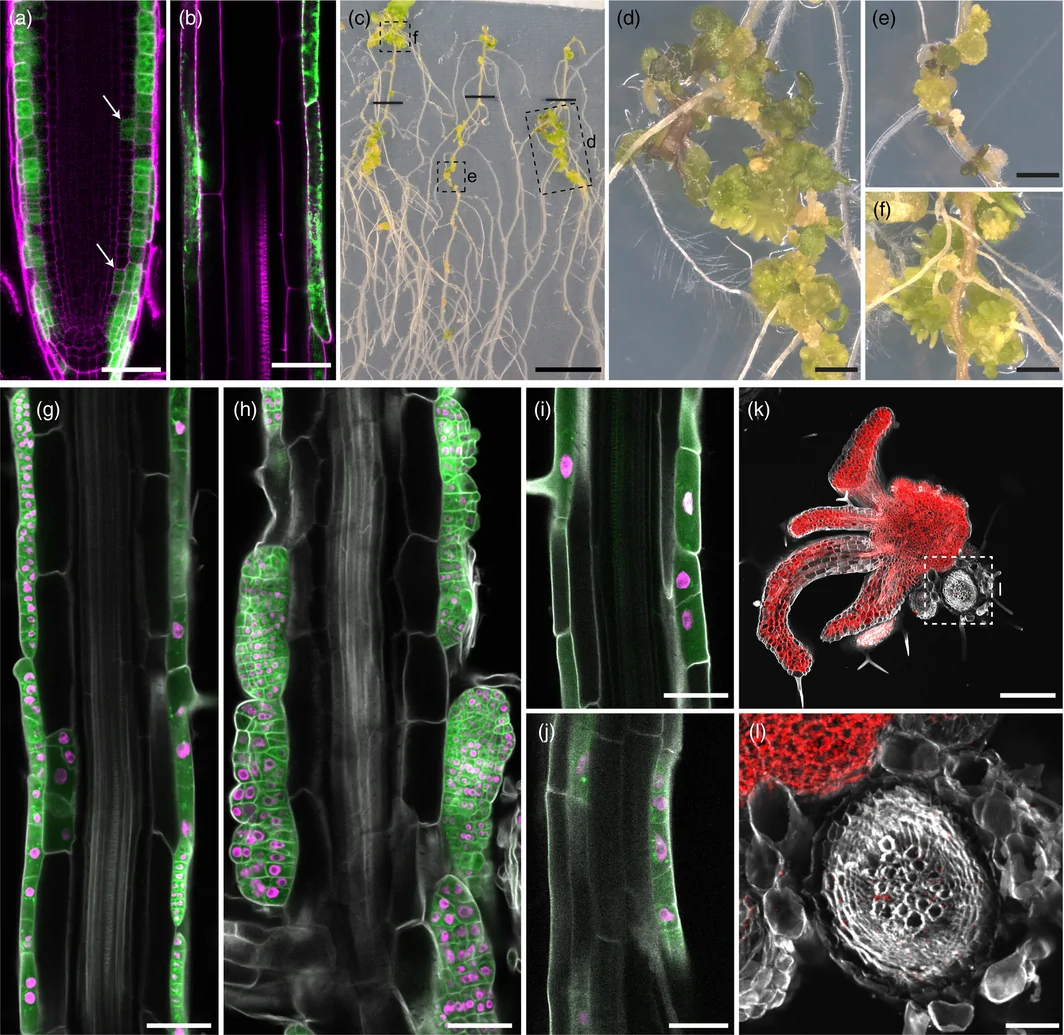
Epidermis‐specific PLT1/WOX5‐induced somatic embryogenesis in TRAC‐IT(pWER>>PLT1/WOX5) line T3‐23. (a, b) Representative images of clone formation in the root meristem (a) and differentiation zone (b) following a 24 h induction on 10 μM DEX. Arrows indicate occasional cortex clones. YFP fluorescence in false green, PI cell wall staining in magenta. (c) Low‐magnification image showing regeneration on roots that were sequentially treated with DEX for 24 h followed by 10 μM β‐Est for 22 days. The position of the root tip at the time of transfer to β‐Est is indicated with a black line. (d–f) Higher magnification images of regenerated shoots and embryos that formed on the part of the root that developed after transfer to β‐Est (d, e, dashed boxes in c) and on lateral roots (f, dashed box in c). (g–j) Representative confocal images of dividing epidermis‐derived clones originating from the meristem (g, h) and the differentiation zone (i, j) after a 24 h induction with DEX, followed by 3 (g, i) or 6 (h, j) days of induction on β‐Est. YFP fluorescence in false green, WOX5‐2xGFP in magenta, SR2200 cell wall staining in grayscale. (k) Representative vibratome cross‐section of regenerated tissues after treatment with DEX for 24 h and β‐Est for 20 days. (i) Higher magnification image of the cross‐section shown in (k) focusing on the primary root (dashed box). Chlorophyll autofluorescence is shown in red, Renaissance SR2200 in greyscale. Scale bars: (a, b, g–j, l) 50 μm; (c) 1 cm; (d–f) 1 mm; (k) 230 μm.

After 20–22 days of β‐Est induction, 38/43 seedlings exhibited regeneration of somatic embryos and shoots from the part of the primary root that developed after transfer to β‐Est, as determined by root tip position marked at transfer (Figure 5c–e). This indicates that meristematic epidermal clones, present at the point of transfer to β‐Est, gave rise to somatic embryos. Although epidermal clones were present in the differentiation zone upon transfer to β‐Est media (Figure 5b), root tip marking at transfer indicated that these did not display somatic embryo formation in any of the examined seedlings. We also observed regeneration from lateral roots in 43/43 seedlings (Figure 5c,f), which corresponded with the activity of the *WER* promoter in late‐stage primordia and emerging lateral roots (Figure S11a,b), as well as the presence of clones following DEX induction (Figure S11c,d). In contrast, substituting the β‐Est incubation for a mock did not induce regeneration in any of the 32 tested seedlings (Figure S12a). Substituting the DEX induction with a mock treatment to allow only non‐induced clone formation, followed by β‐Est induction resulted in occasional regeneration events in 5/16 seedlings (Figure S12b). For both controls, the proportion of roots showing regeneration is significantly decreased compared with the DEX/β‐Est‐treated seedlings (Table S2), supporting the notion that regeneration results from PLT1/WOX5 induction within clones.

To confirm that the regeneration originates from epidermal clones using *TRAC‐IT(pWER>>PLT1/WOX5)*, we subjected eight regenerating roots to confocal analysis at 3 or 6 days after transfer to β‐Est. In line with the observed clones after DEX induction (Figure 5a,b), after 3–6 days of β‐Est induction, we did not observe clones outside of the targeted LRC and epidermis tissues apart from the previously reported occasional cortex clones (Figure 5g–j; Table S2). In none of the eight roots did we detect pericycle clones. We could clearly detect clone‐specific fluorescence from accumulated PLT1‐3xmCherry and WOX5‐2xGFP in all tested roots at all timepoints (Figure S13a–d, Table S2). In addition, we observed a high degree of cell proliferation, including periclinal divisions, in meristematic clones at 3 to 6 dpi (Figure 5g,h; Table S2). Comparable levels of proliferation were observed in clones generated in lateral roots (Figure S11e,f; Table S2). In contrast, epidermal clones that formed in the differentiation zone produced only a few, mostly anticlinal, divisions at 3 and 6 dpi (Figure 5i,j; Table S2). Besides lateral root formation and secondary vascular development, both independent of clone induction, we did not observe any notable cell proliferation outside of clones (Figure 5g–j; Table S2). This indicates that the induced regeneration is autonomous to the clones. We substantiated this by performing confocal analysis on 13 independent embryonic structures that had regenerated 18 days after transfer to β‐Est. YFP fluorescence was detected throughout all embryos (Figure S14a,b; Table S2), confirming their clonal origin. To further investigate the cellular origin of regenerated embryos and shoots derived from the primary root meristem clones, we performed confocal analysis at 20 dpi of β‐Est using vibratome cross‐sections of regions that displayed regeneration. In all cases (*n* = 5), regenerated embryos and shoots originated from the outer layers of the root (Figure 5k,l; Table S2), in agreement with somatic embryogenesis originating from induced epidermal clones.

Finally, to exclude potential line‐specific effects, we repeated our analysis on the second best‐regenerating homozygous line (T3‐21). We found that this line also recapitulates the phenotypes observed for line T3‐23 (Figure S15), supporting that the observed behavior of the TRAC‐IT system is robust across independent lines.

Together, these results show that TRAC‐IT derived tissue‐specific clones can provide insight in the role of cell type and developmental (st)age during PLT1/WOX5‐induced somatic embryogenesis, and that tissue‐specific transgene induction can be sustained independently of ongoing reprogramming and cell division.

## DISCUSSION

In this work, we report the design and functionality of the Tissue‐specific Recombination‐Activated Clonal and Inducible Transcription system (TRAC‐IT). In TRAC‐IT, a DEX‐inducible and tissue specifically expressed CRE recombinase (CREi‐GR) creates erYFP‐marked clones, that constitutively express *XVE* to allow subsequent β‐Est inducible clone‐specific transgene expression. By uncoupling transgene expression from the original tissue‐specific promoter, TRAC‐IT enables sustained tissue‐specific gene induction, even when cells within clones divide and/or undergo reprogramming.

The CRE/*loxP* module has previously been used in studies aiming to test autonomy of gene activity or the effect of local gene activation during development. There, the clonal transgene was either directly activated by placing it directly downstream of a promoter‐driven *loxP*‐flanked stuffer (Ishida et al., 2016), or transactivated via a *GAL4VP16* (*GV*) positioned downstream of the *loxP*‐flanked stuffer (Heidstra et al., 2004). Although the *GV* transactivation approach allows for the activation of multiple transgenes, neither strategy allows for temporal control of clonal transgene expression. In contrast, by using the inducible XVE/*LexA* transactivation system within clones, TRAC‐IT represents a significant technical advance by enabling temporal control over the activation (and termination) of expression of multiple genes. In addition, the XVE/*LexA* transactivation system provides control of the gene induction strength by adjustment of β‐Est concentration (Zuo et al., 2000). Besides the activation of functional transgenes, the CRE/*loxP* module is widely used for lineage tracing by placing a marker gene downstream of a constitutive promoter‐driven *loxP*‐flanked stuffer cassette, leading to stably marked clone formation upon recombination (Efroni et al., 2016; Zhai & Xu, 2021). This principle is integrated in TRAC‐IT, as the P2A peptide allows for constitutive expression of both *XVE* and *erYFP* within clones. Hence, TRAC‐IT does not only allow for temporal and quantitative control over transgene expression within tissue‐specific clones, but also for tracing the lineage of these clones irrespective of fate change.

We have demonstrated that TRAC‐IT can reliably activate clone formation. In all tested lines, clone formation was largely tissue‐specific. The observation of some non‐tissue‐specific clones is in line with a previous study that also used *CRE‐GR* for marked clonal gene activation (Wachsman et al., 2011). This indicates that the formation of non‐tissue‐specific clones is inherent to the use of a tissue‐specific *CRE‐GR*. In both this and the previous study, non‐specific clones were generally found in directly adjacent tissue layers. This hints at movement of either *CREi‐GR* mRNA or encoded protein, although the latter is unlikely given the binding of the GR domain to cytosolic heat shock protein complexes (Lockett et al., 2024). Alternatively, non‐tissue‐specific clones might be caused by low activity of the chosen promoters in these adjacent tissues. Besides non‐tissue‐specific clones, occasional non‐induced clones were detected in mock‐treated roots of all our tested lines. As all non‐induced clones were observed within the targeted tissues, this suggests that at the levels present in target cells, the CREi‐GR protein exhibits a small degree of nuclear activity in the absence of DEX, which has also been reported for other GR–protein fusions (Yamaguchi et al., 2015).

Although the frequency of non‐tissue‐specific and non‐induced clones is low compared with specific, induced clones, these events represent a limitation of the TRAC‐IT system. This may present an issue when coupled to strong developmental regulators, where minimal misexpression could lead to unintended phenotypic consequences. Hence, it is important to implement proper controls regarding the formation and development of clones over time, as well as the selection of a tight tissue‐specific promoter during experimental design. Furthermore, although not observed in any of the lines in this study, non‐induced clone formation in germline cells will lead to progeny with constitutive clone identity. Given the correlation of the sporadic non‐induced clones being formed in the tissue marked by the applied promoter in our study, the likelihood of these events will depend on the activity domain of the promoter used to drive *CREi‐GR* expression.

In addition to demonstrating that DEX induction induces clone formation, we have found that the number of clones formed is tunable through both the duration and concentration of the DEX application. Depending on the experimental needs, the DEX induction parameters may be optimized, as they could vary with experimental conditions, the tissue‐specific promoter, and the transgenic line used.

Using fluorescent marker genes, we showed that the erCFP, PLT1‐3xmCherry, and WOX5‐2xGFP proteins accumulated exclusively in clones. However, the observation of mCherry mobility highlights the importance of restricting intracellular movement of the induced genes if their effect needs to be studied cell autonomously. Increasing protein mass by attachment of double or triple‐fluorophore tags, as applied in *TRAC‐IT(pWER>>PLT1/WOX5)* incorporating *PLT1‐3xmCherry* and *WOX5‐2xGFP*, is one way to restrict protein trafficking, provided that such a change in size does not affect protein activity. A beneficial side effect of this approach is the opportunity to visualize transgene expression with a fluorescence microscope.

TRAC‐IT is fully compatible with Golden Gate cloning and the MoClo Toolkit (Weber et al., 2011; Werner et al., 2012), and thus easily adapted to incorporate (tissue‐specific) promoters and transgenes of interest. The application of the intronized *CREi‐GR* gene prevents premature recombination in bacteria, enabling all components to be contained in a single T‐DNA vector and eliminating the need for sequential transformation or crossing. Despite the size of the constructs and the number of transgenes, we did not encounter any signs of silencing over the generations of our TRAC‐IT lines, some of which have been propagated to T4. Although we only show the application of TRAC‐IT in Arabidopsis roots, we expect similar functionality in other organs and plant species, given the wide successful utility of the *CRE‐GR* and XVE/*LexA* modules (Ishida et al., 2016; Kareem et al., 2022; Sugano et al., 2018; Tameshige et al., 2013; Zhang et al., 2024). However, some plant species with high phytoestrogen levels such as the model legume *Medicago truncatula* may show high background activity of XVE (Liu & Yoder, 2016). Substituting XVE for an STF based on the insect ecdysone receptor, which is less widely used but nonetheless functional in plants (Tavva et al., 2007), could offer a solution for such species. The Golden Gate‐based modularity of TRAC‐IT further enables the use of alternative constitutive promoters in Module II when desired. While the *AtUBQ10* promoter successfully supported the induction of fluorescent proteins and PLT1/WOX5‐driven regeneration in this study, previous reports indicate that its expression is not completely homogeneous between root cell types (Krogman et al., 2020). Depending on the required expression characteristics, the choice of constitutive promoter may be adjusted accordingly.

To highlight the ability of TRAC‐IT to sustain tissue‐specific gene induction even after cell division and reprogramming, we used it to induce *PLT1*/*WOX5*‐mediated somatic embryogenesis in epidermal cells of the Arabidopsis root. Although clones were formed in both the root meristem and differentiation zone, strong cell proliferation and regeneration were only observed from meristem‐derived epidermal clones. This aligns with observations on cell proliferation during early stages of constitutive *PLT1/WOX5* overexpression (Wittmer et al., 2025). It shows that epidermal cells in the root meristem display embryogenic capacity in response to PLT1/WOX5, while epidermal cells in the differentiation zone do not. Although the involvement of the occasional cortical clone in the regeneration cannot be completely excluded at this stage, the lack of detectable clones in the pericycle and the clonal origin of regenerated embryos show that regeneration competence is not restricted to the pericycle. Furthermore, cell divisions were not induced outside of clones, showing that regeneration derived from dividing clones does not require co‐activation of regenerating pericycle. In this sense, TRAC‐IT‐mediated *PLT1/WOX5*‐induced regeneration breaks the paradigm for hormone‐mediated regeneration from Arabidopsis root tissues (Atta et al., 2009; Zhang et al., 2022). Although out of the scope of the current study, a systematic and cell‐type‐resolved comparison of regeneration competence, including the extent of off‐target contributions, remains an important goal for future studies on PLT1/WOX5‐mediated regeneration. Notably, the constitutive accumulation of erYFP in TRAC‐IT clones allows the isolation of these marked cells by fluorescence‐activated cell sorting (Bargmann & Birnbaum, 2010), providing novel experimental opportunities to unravel the mechanisms that underly embryogenic potential.

Taken together, TRAC‐IT is a versatile, easily adaptable tool for tissue‐specific, clone‐based inducible transgene expression that provides new technical opportunities to explore the mechanisms underlying plant development.

## METHODS

### Plant growth conditions

All *in vitro* plant growth and experiments with TRAC‐IT were performed on ½ GM media as described (Wittmer et al., 2025).

### Cloning

All plasmids were constructed by Golden Gate cloning using the MoClo Toolkit (Addgene Kit #1000000044) and Plant parts (Addgene Kit #1000000047) (Engler et al., 2014; Weber et al., 2011; Werner et al., 2012). Primers to amplify sequences for cloning are listed in File S3. Fragments containing internal BpiI and/or BsaI recognition sites were amplified in multiple segments (primers numbered accordingly), with silent mutations introduced in the primers to eliminate these sites.

#### Common parts

To create *pICH41274‐ADHTer*, 250 bp downstream of the *ADH* (*AT1G77120*) coding sequence was amplified from Col‐0 genomic DNA using *ADHTer_F/R* and restriction/ligated into *pICH41276*. To create *pICH41308‐erYFP*, the sequence of *erYFP* was amplified from a pre‐existing clone using *erYFP_l0F/R* and cloned into *pICH41308*.

#### Reporter constructs

Promoter sequences were amplified from Col‐0 genomic DNA and cloned into either *pICH41233* (*pSHR*, *pWOX5*) or *pICH41295* (*pWER, pXPP, pSCR, pCO2B*).

To create the lvl‐2 binary construct for marker expression of *pSCR::erYFP*, a lvl‐1 construct was first generated by combining *pICH41295‐pSCR* with *pICH41308‐erYFP* and *pICH41276‐ADHTer* into *pICH47742*. Subsequently, the final lvl‐2 construct was generated by combining *pICH47732‐FASTR* (Kerstens et al., 2021) with *pICH47742‐pSCR::erYFP* and the *pICH41744* end linker into *pICSL4723*.

To create the erGFP‐based reporters, we first generated the lvl‐2 *FASTR‐BsaILacZ::erGFP* binary acceptor plasmid by combining *pICH47732‐FASTR*, a PCR fragment harboring a *BsaI*‐flanked *lacZ* cassette (amplified from *pICH47742* using primers *lacZ_acc_F/R*), a PCR fragment harboring *erGFP‐NosT* (amplified from a pre‐existing lvl‐1 construct containing *erGFP* (Sabatini et al., 2003) using primers *erGFP‐NosT_acc_F/R*), and the *pICH41744* end linker into *pICSL4723*. The level‐0 *pICH41295*‐promoter vectors were used to directly clone individual promoters into this acceptor to create the final lvl‐2‐1 plasmids, replacing the *LacZ* cassette upstream of the *erGFP* gene. For the *pICH41233‐*promoter vectors, we combined these with *pICH41402* (TMV Omega leader) in the reaction to generate lvl‐2‐1 vectors.

#### TRAC‐IT fluorophore constructs

The original, non‐intronized *CRE‐GR* sequence was amplified from *pENTR221‐CRE:GR* (Wachsman et al., 2011) using *CREGR0_F/R* primers and cloned to create *pICH41308‐CRE:GR*. We generated an intronized *CREi‐GR* version, in which the third intron from the *AT1G06220* gene was inserted between the AG and GC residues at nucleotide position 237 of the *CRE* sequence according to Harrington et al. (2020). To introduce this intron, the *CRE‐GR* sequence was amplified in two parts with *CREGR1_F/R* and *CREGR2_F/R*. The intron was amplified from *pICSL80016* (containing a GUS gene with an intron of *AT1G06220*) using *CREintron_F/R*. The three fragments were then cloned together to create *pICH41308‐CREi‐GR*. The lvl‐0 tissue‐specific promoter constructs were then combined with *pICH41308‐CREi‐GR* and *pICH41276‐ADHTer* into *pICH47742* to generate lvl‐1 constructs of module I.

The *AtUBQ10* promoter was amplified from *pICSL12015* using *pATUBQ10_1251F/R* and cloned to generate *pAGM1251‐pAtUBQ10*. To create the *loxP‐CRT1‐loxP* cassette, two *loxP* sites with 4‐basepair overhangs were assembled by annealing 10 μM single‐stranded oligos *lox1F/lox1R* and *lox2F/lox2R*. The *CRT1* gene (encoding a phytoene desaturase conferring norflurazon resistance; Misawa et al., 1993) with *Nos* terminator was amplified from *pCB1* (Heidstra et al., 2004) using primers *CRT1_l0F/R*. The two *loxP* sequences and the *CRT1* PCR fragment were Golden Gate cloned to create *pAGM1276‐loxP‐CRT1‐NosT‐loxP*. To create the *XVE‐P2A‐erYFP* fusion, the three coding sequences were amplified with *XVE‐l0_F/R*, *P2A‐l0_F/R*, and *erYFP‐l0_F/R* primer pairs from *pICH41308‐XVE* (Wittmer et al., 2025), *pICH47732‐RUBY* (Wittmer et al., 2025) and *pICH41308‐erYFP*, respectively. Each PCR fragment was cloned into the pAGM1311 level‐1 acceptor, which was subsequently combined to generate *pICH41308‐XVE‐P2A‐erYFP*. Combining *pAGM1251‐pAtUBQ10*, *pAGM1276‐loxP‐CRT1‐NosT‐loxP*, *pICH41308‐XVE‐P2A‐erYFP*, and *pICH41276‐AtUBQ10Ter* into *pICH47752* then generated the lvl‐1 construct of Module II.

To create level‐0 plasmids for *ermCherry* and *erCFP*, we amplified the cloning vector harboring the N‐terminal ER signal peptide and the C‐terminal ER retention signal of *pICH41308‐erYFP* with *erFluoBB_F/R*. We then amplified the *mCherry* and *CFP* coding sequences with *mCherry_erF/R* and *CFP_erF/R* from pre‐existing clones. The amplified vector backbone and fluorophore sequences were Golden Gate cloned to create *pICH41308‐ermCherry* and *pICH41308‐erCFP*. Lvl‐1 plasmids of Module III were then created by combining *pICH41295‐pLexA* with *pICH41308‐ermCherry and pICH41276‐HSPTer* (Wittmer et al., 2025) into *pICH47761* to form *pICH47761‐pLexA::ermCherry*, and the combination of *pICH41295‐pLexA* with *pICH41308‐erCFP* and *pICH41414‐T35S* into *pICH47772* generated *pICH47772‐pLexA::erCFP*.

Finally, the *TRAC‐IT(pWER>>mCherry/CFP)*, *TRAC‐IT(pSCR>>mCherry/CFP)*, and *TRAC‐IT(pXPP>>mCherry/CFP)* constructs were generated by combining *pICH47732‐pNos::BAR* (Wittmer et al., 2025), the lvl‐1 plasmid of module I with the respective promoter, the lvl‐1 plasmid of Module II, both lvl‐1 plasmids of Module III, and the *pICH41800* end linker into *pICSL4723*.

#### Reporter‐clone constructs for testing tissue‐specific clone formation with characterized promoters

*pAGM1251‐pAtUBQ10*, *pAGM1276‐loxP‐CRT1‐NosT‐loxP*, and *pICH41276‐AtUBQ10Ter* (see above) were combined with *pICH41308‐erGFP* (Wang et al., 2024) into *pICH47732* to create the level 1 construct for clone‐specific erGFP expression. This construct was subsequently combined with each of the Level 1 constructs for tissue‐specific expression of *CREi‐GR* (TRAC‐IT Module I, see above) and the *pICH41744* end linker into *pICSL4723* to yield the final lvl‐2 *CREi‐GR>>loxP‐erGFP* constructs.

#### TRAC‐IT construct for generation of somatic embryos from epidermal cells

The sequence of *3xmCherry* was amplified from *pGGC026* (Lampropoulos et al., 2013) using *3xmCherry_F/R1* and *3xmCherry_F/R2* and cloned to generate *pAGM1301‐3xmCherry*. The *SG15* linker sequence was synthesized as a linear fragment (Twist Biotech) and cloned to create *pAGM1311‐SG15*. The sequence of *2xGFP* was amplified from a pre‐existing *3xGFP* clone using *2xGFP_F/R* and Golden Gate cloned together with *pAGM1311‐SG15* to generate *pAGM1301‐SG15‐2xGFP*. Lvl‐1 plasmids of Module III were then created by combining *pICH41295‐pLexA* with *pAGM1287‐PLT1* (Wittmer et al., 2025), *pAGM1301‐3xmCherry*, *and pICH41276‐HSPTer* into *pICH47761* to form *pICH47761‐pLexA::PLT1‐3xmCherry*, and the combination of *pICH41295‐pLexA* with *pAGM1287‐WOX5* (Wittmer et al., 2025), *pAGM1301*‐*SG15‐2xGFP*, and *pICH41414‐T35S* into *pICH47772* generated *pICH47772‐pLexA::WOX5‐SG15‐2xGFP*.

Next, the *TRAC‐IT(pWER>>PLT1/WOX5)* construct was generated by combining *pICH47732‐pNos::BAR*, the lvl‐1 plasmid of module I containing the WER promoter, the lvl‐1 plasmid of Module II, the lvl‐1 Module III plasmids *pICH47761‐pLexA::PLT1‐3xmCherry* and *pICH47772‐pLexA::WOX5‐SG15‐2xGFP*, and the *pICH41800* end linker into *pICSL4723*.

#### Constructs for Nicotiana infiltration

*pICH41308‐erYFP*, *pICH41308‐XVE*, or *pICH41308‐XVE‐P2A‐erYFP* was combined with *pICH51277* (*p35S::TMV Ω*) and *pICH41421* (*NosT*) into *pICH47742* to respectively generate the *pICH47742‐p35S::erYFP*, *pICH47742‐p35S::XVE*, and *pICH47742‐p35S::XVE‐P2A‐erYFP* lvl‐1 plasmids. The *pICH47742‐pLexA::erCFP* lvl‐1 plasmid was created by combining *pICH41295‐pLexA*, *pICH41308‐erCFP*, and *pICH41421* into *pICH47742*. Subsequently, each lvl‐1 plasmid was individually cloned into lvl‐2 by combining them with the *pICH54011* dummy and *pICH41744* end linker into *pICSL4723*. This respectively yielded the final *p35S::erYFP*, *p35S::XVE*, *p35S::XVE‐P2A‐erYFP*, and *pLexA::erCFP* plasmids.

### Plant transformation

Arabidopsis plants were transformed with *Agrobacterium tumefaciens* strain C58C1.pMP90 (Koncz & Schell, 1986), using the floral dip method (Clough & Bent, 1998). Copy number analysis was performed on T1 plants by droplet digital PCR (ddPCR) using Taqman probes. *AAP1* (AT1G58360) was used as a reference gene. Primer and probe sequences are listed in File S3.

For infiltration of *N. benthamiana* leaves, *Agrobacteria* were inoculated from plate into a 5 mL liquid LB culture supplemented with 50 mg L^−1^ kanamycin and 30 mg L^−1^ rifampicin, and grown for 2 days at 28°C. This culture was diluted 1:100 into LB with antibiotics, 10 mM MES (pH 5.6) and 40 μM acetosyringone, and grown overnight. Bacteria were pelleted (3200× ***g***, 10 min) and resuspended in 10 mM MgCl_2_ with 200 μM acetosyringone to an OD600 of 0.5. An *Agrobacterium* culture carrying the anti‐silencing *P19* helper plasmid was grown and resuspended in the same fashion. Cultures were kept at room temperature without shaking for 3 h, after which they were mixed 1:1 (*p35S::erYFP* or *p35S::XVE‐P2A‐erYFP*: P19) or 1:1:1 (*p35S::erYFP* or *p35S::XVE‐P2A‐erYFP*: *pLexA::erCFP*: P19). With these mixtures, the Nicotiana leaves were infiltrated as described (Díaz‐Triviño et al., 2017). Four days after infiltration, leaves were analyzed by confocal microscopy. Solutions for gene induction were prepared by supplementing tap water with either 10 μM β‐Estradiol or the equal amount of DMSO solvent for the mock treatment. These solutions were infiltrated in the same fashion, 1 day prior to analysis.

### Protein extraction and Western blot

#### Nicotiana

Three days after infiltration, each harvested Nicotiana leaf was flash‐frozen in liquid nitrogen and ground using a mortar and pestle. Around 0.5 ml of the ground‐up powder was measured in an Eppendorf tube, to which 750 μl of RIPA buffer (Huisman et al., 2020) was added. Samples were gently mixed until all powder was resuspended and incubated on ice for 30 min with gently tumbling from time to time. Samples were centrifuged for 10 min at 15 000× ***g***, after which the supernatant was collected for further analysis. A total of 25 μL of the supernatant was gently mixed with 25 μl of 2× SDS‐buffer (Huisman et al., 2020) and incubated at 95°C for 10 min. Of this, 20 μl was loaded onto a precast 4–12% polyacrylamide Mini‐PROTEAN TGX gel (Bio‐Rad), which was run according to the manufacturer protocol. Proteins were transferred to a PVDF membrane using the Bio‐Rad Trans‐Blot turbo system. The blot was blocked in PBST (8 g L^−1^ NaCl, 0.2 g L^−1^ KCl, 1.44 g L^−1^ Na_2_HPO_4_, 0.24 g L^−1^ KH_2_PO_4_, 0.3% Tween‐20) + 3% BSA for 1 h, briefly washed in PBST + 1% BSA, incubated with αGFP‐HRP (Milteny Biotec) at 1:5000 in PBST + 1% BSA for 1 h at room temperature, washed 3× in PBST + 1% BSA for 20 min, and briefly washed in PBS (PBST without Tween‐20). After this, the blot was developed with the Immun‐Star™ HRP Chemiluminescent Kit (Bio‐Rad) according to the manufacturer protocol and chemiluminescence was imaged on a Gel Doc XR+ (Bio‐Rad).

#### Arabidopsis

Seedlings were germinated and grown on 1/2GM with 10 μM DEX for 8 days to maximize clone formation. Plant material was harvested into 2 ml tubes containing a stainless‐steel ball and frozen in liquid nitrogen. Ten whole roots were harvested for both TRAC‐IT lines and Col‐0 wild‐type, whereas 40 root tips (~3 mm) were harvested for pCO2B::erGFP to increase the relative GFP content. Tissue was homogenized using a TissueLyser LT (Qiagen) at 50/s for 1 min. A total of 200 μL of 2× SDS‐buffer (Huisman et al., 2020) was added to the samples, which were vortexed for 30 s, incubated at 99°C for 15 min, and centrifuged at maximum speed for 3 min. The supernatant was taken and 20 μl was loaded on a precast 12% polyacrylamide Mini‐PROTEAN TGX gel (Bio‐Rad). Samples of pCO2B::erGFP and Col‐0 were not diluted; samples of TRAC‐IT(pWER>>mCherry/CFP) T3‐18 and TRAC‐IT(pSCR>>mCherry/CFP) T3‐11 were respectively 4× and 10× diluted in 2xSDS‐buffer to equalize GFP input. Electrophoresis, blotting, and detection were performed as described for Nicotiana.

### Confocal imaging

All confocal imaging was performed on a Zeiss LSM710, unless stated otherwise.

#### Reporter lines

For the analysis of *WER* promoter expression, we used a line from previous work (Willemsen et al., 2008). The other promoters were cloned with either *erGFP* or *erYFP* (see above) and transformed into Arabidopsis.

All reporter lines were imaged in roots of live seedlings using propidium iodide (PI) staining (van den Berg et al., 1995). Excitation of CFP was performed at 458 nm, GFP and YFP at 488 nm. In the case of CFP, a laser at 543 nm was used in parallel to excite PI. Detection of CFP at 463–568 nm, GFP at 493–551 nm, YFP at 517–542 nm, and PI at 584–718 nm.

#### Transiently transformed Nicotiana leaves

Transformed 1 × 1 cm leaf sections were cut off and submerged in tap water on the slides, with the abaxial side facing the cover slip. CFP was exited at 458 nm and detected at 463–520 nm. YFP was excited in a separate track at 514 nm and detected at 520–735 nm.

#### Analysis of clone formation

For TRAC‐IT T3 lines, roots were fixed in 4% PFA in 50 mM PIPES (pH 7.0) by means of vacuum‐infiltration for 1 h followed by overnight incubation at 4°C. Subsequently, roots were washed with 50 mM PIPES, transferred to ClearSeeAlpha solution (Kurihara et al., 2021) supplemented with 0.1% Renaissance SR2200 (Musielak et al., 2015) and incubated in the dark, while softly shaking at room temperature for 3–7 days. After incubation, roots were imaged using separate tracks for SR2200 (excited at 405 nm, recorded at 410–507 nm) and YFP (excited at 514 nm, recorded at 519–620 nm). For quantification of clone cells within z‐stacks, counting was performed by hand using the ortho feature in Zeiss Zen 3.10. Roots that had undergone non‐induced clone formation in a stem cell prior to DEX induction and therefore possessed a clone consisting of an entire cell file (*n* = 3/126) were excluded from the analysis. Clone formation on TRAC‐IT(pWER>>PLT1/WOX5) lines was imaged on live roots using PI staining. Excitation was performed at 514 nm, detection of YFP at 493–551 nm, detection of PI at 607–718 nm. Clone formation on CREi‐GR>>loxP‐erGFP lines was analyzed on a Leica TCS SP8 HyD using PI staining. Excitation was performed at 488 nm, detection of GFP at 490–550 nm, detection of PI at 590–650 nm. Clone analysis on 27 TRAC‐IT T2 lines to confirm tissue specificity following DEX and Mock treatments was performed on a Zeiss LSM900, using ClearSeeAlpha with SR2200 as described above. In separate tracks, SR2200 was excited at 405 nm and detected at 400–600 nm, while YFP was excited at 488 nm and detected at 410–546 nm.

#### Analysis of erCFP and ermCherry induction in clones

Live roots of TRAC‐IT(pWER>>mCherry/CFP), TRAC‐IT(pSCR>>mCherry/CFP), and TRAC‐IT(pXPP>>mCherry/CFP) lines were imaged immersed in tap water without staining to optimize detection of fluorescent proteins. To avoid crosstalk, each fluorophore was imaged in separate tracks with strict detection ranges. CFP was excited at 458 nm and detected at 463–507 nm; YFP was excited at 514 nm and detected at 519–590 nm; mCherry was excited at 543 nm and detected at 578–696 nm. A transmitted light channel (PMT Trans) was added to the YFP track to allow for visualization of the root outline.

#### Analysis of regeneration in TRAC‐IT (pWER>>PLT1/WOX5) lines

Induced root tissue was harvested at the desired timepoints, and fixing and clearing was performed as described above, with the alteration that 0.1% Renaissance SR2200 was not supplemented to the initial ClearSeeAlpha solution. One day prior to analysis, the ClearSeeAlpha solution was refreshed, now also adding 0.1% Renaissance SR2200. After this, roots were imaged using one track for SR2200 fluorescence (excited at 405 nm, detected at 410–507 nm) and YFP fluorescence (excited at 514 nm, detected at 519–620 nm), and one track for GFP fluorescence (excited at 488 nm, detected at 490–513 nm) and mCherry fluorescence (excited at 543 nm, detected at 596–691 nm). The 405 nm laser was used at a relatively low intensity to minimize detection of SR2200 fluorescence in the YFP channel. For the imaging of regenerated somatic embryos, each fluorophore was imaged in a separate track to minimize crosstalk.

#### Sectioning of regenerated tissue

Regenerated tissue was fixed in 4% PFA in 50 mM PIPES (pH 7.0) by means of vacuum‐infiltration for 1 h, and incubation at 4°C for at least 3 days. Tissues were washed in 50 mM PIPES, after which they were suspended in 800 μl molten 6% low‐melt agarose at 37°C in a 2 ml round‐bottom tube. The agarose was solidified by transferring the tube to ice for 10 min, after which the agarose block was removed from the tube and sectioned into 80 μm slices on a Leica VT1000 S vibratome. The tissue slices were immersed in PBS (pH 7.2) supplemented with 0.1% Renaissance SR2200 on the microscope slides and imaged on a confocal microscope using a 405 nm laser for SR2200 (detected at 410–585 nm), and a 633 nm laser for chlorophyll fluorescence (detected at 647–721 nm).

#### Other imaging

Close‐up images of regenerated tissues were acquired on a Zeiss Stereo Discovery‐V12 microscope equipped with a Plan S 1.0 lens and an Axiocam MRc5 camera. Epifluorescence images of seedlings constitutively expressing erYFP were acquired on a Leica M165 FC microscope equipped with a Leica DFC450 C camera.

## AUTHOR CONTRIBUTIONS

TW, JW, HN, BS, and RH conceptualized the research; TW and RW performed research; JW and TB performed preliminary research and generated constructs; AvZ performed analysis on the generated TRAC‐IT lines; TW, BS, and RH wrote the paper.

## CONFLICT OF INTEREST

The authors declare no conflict of interest.

## Supporting information


**File S1.** Binocular images of TRAC‐IT roots treated with DEX or Mock.

**File S2.** Clone counts from TRAC‐IT induction assays.

**File S3.** Oligonucleotide sequences.

**Figure S1.** Production of separate XVE and erYFP proteins from the *XVE‐P2A‐erYFP* fusion gene.
**Figure S2.** Marker lines showing the expression pattern of six experimentally validated tissue‐specific promoters in the Arabidopsis root.
**Figure S3.** Intronization of *CRE‐GR* prevents recombination of *TRAC‐IT* plasmids in *Agrobacterium*.
**Figure S4.** The *CREi‐GR/LoxP‐CRT1‐LoxP* recombination system used in TRAC‐IT generates cell‐type‐specific clones.
**Figure S5.** Increase in clone‐derived YFP fluorescence in TRAC‐IT roots following DEX application.
**Figure S6.** Relationship between T‐DNA copy number (x‐axis) and the percentage of root sections displaying non‐induced clones (y‐axis) across all 30 analyzed TRAC‐IT(pWER>>mCherry/CFP), TRAC‐IT(pSCR>>mCherry/CFP) and TRAC‐IT(pXPP>>mCherry/CFP) T2 lines.
**Figure S7.** Tissue specificity of clone formation following 24 h induction with 10 μM DEX in the remaining 27 TRAC‐IT lines.
**Figure S8.** Tissue specificity of non‐induced clone formation following 24 h induction with a mock in the remaining 27 TRAC‐IT lines.
**Figure S9.** Representative confocal image showing autofluorescence in the CFP channel in the root meristem of wild‐type Col‐0 seedlings, recorded at the same settings as the root meristem images of TRAC‐IT(pSCR>>mCherry/CFP) line T2‐26 seedings in Figure 4.
**Figure S10.** Free mCherry signal in cells surrounding clones.
**Figure S11.** Clone‐derived regeneration from lateral roots of TRAC‐IT(pWER>>PLT1/WOX5) line T3‐23 seedlings.
**Figure S12.** Representative images showing the reduced regeneration phenotype in mock‐treated TRAC‐IT(pWER>>PLT1/WOX5) line T3‐23 seedlings.
**Figure S13.** Clone‐specific accumulation of PLT1‐3xmCherry and WOX5‐2xGFP in TRAC‐IT(pWER>>PLT1/WOX5) line T3‐23.
**Figure S14.** Developing embryonic structures following induction of *PLT1* and *WOX5* originate from clones.
**Figure S15.** Epidermis‐specific PLT1/WOX5‐induced regeneration in TRAC‐IT(pWER>>PLT1/WOX5) line T3‐21.

**Table S1.** Performance of 30 independent T2 lines carrying *TRAC‐IT(pWER>>mCherry/CFP)*, *TRAC‐IT(pSCR>>mCherry/CFP)*, or *TRAC‐IT(pXPP>>mCherry/CFP)*, assessed by fluorescence microscopy.

**Table S2.** Quantification of observed phenotypes in regeneration‐related experiments performed on TRAC‐IT(pWER>>PLT1/WOX5) T3‐23.

## Data Availability

The data that supports the findings of this study are available in the Supplementary Material of this article.
