# Supplementary material for: Tissue‐specific recombination‐activated clonal and inducible transcription (TRAC‐IT) enables controlled gene activation in stably marked tissues
Source: Plant J. 2026 Sep 28;127(6):e71151. doi: 10.1111/tpj.71151 (PMC13617364; doi:10.1111/tpj.71151)
Supplement: Supplementary file 1 [file TPJ-127-0-s002.docx]

**Table S2:** Quantification of observed phenotypes in regeneration-related experiments performed on TRAC-IT(pWER>>PLT1/WOX5) T3-23. Statistical analysis on the number of regenerated seedlings was performed against the 1d DEX -> 20-22d β-Est treatment using the chi-square test.

| **Quantification of regenerated seedlings** | | |
| --- | --- | --- |
| Treatment | # of regenerated seedings | p-value |
| 1d DEX -> 20-22d β-Est | 43/43 | N.A. |
| 1d DEX -> 21d mock | 0/32 | < 0. 000001 |
| 1d mock -> 21d β-Est | 5/16 | < 0. 000001 |
| **Quantification of phenotypes observed after 1d DEX -> 3-6d β-Est** | | |
| Phenotype observed by confocal microscopy | # of analysed roots | |
| Clones in targeted Epidermis/LRC tissues | 8/8 | |
| Occasional clones in cortex | 8/8 | |
| Clone(s) in pericycle | 0/8 | |
| Clone-specific accumulation of PLT1-3xmCherry and WOX5-2xGFP | 8/8 | |
| Proliferation in meristematic clones | 8/8 | |
| Proliferation in lateral root clones | 8/8 | |
| Occasional division in differentiation zone clones | 8/8 | |
| Cell division outside of clones (except for lateral root formation and secondary development) | 0/8 | |
| **Quantification of phenotypes observed after 1d DEX -> 13d β-Est** | | |
| Phenotype observed by confocal microscopy | # of analysed embryonic structures | |
| YFP-fluorescence throughout embryonic structure | 13/13 | |
| **Quantification of phenotypes observed after 1d DEX -> 20d β-Est** | | |
| Phenotype observed by confocal microscopy | # of analysed regeneration cross-sections | |
| Regenerated embryos/shoots from outer root layers | 5/5 | |
| Regenerated embryos/shoots derived from pericycle | 0/5 | |
