# Supplementary material for: Tissue‐specific recombination‐activated clonal and inducible transcription (TRAC‐IT) enables controlled gene activation in stably marked tissues
Source: Plant J. 2026 Sep 28;127(6):e71151. doi: 10.1111/tpj.71151 (PMC13617364; doi:10.1111/tpj.71151)
Supplement: Supplementary file 2 [file TPJ-127-0-s003.docx]

**Figure S1:** Production of separate XVE and erYFP proteins from the *XVE-P2A-erYFP* fusion gene. **a,b** Confocal images of erYFP fluorescence in Nicotiana leaves, 4 days after transient transformation with *35S::erYFP* (**a**) or *35S::XVE-P2A-erYFP* (**b**). YFP fluorescence is shown in false yellow, transmitted light in greyscale. **c.** Western blot performed with anti-GFP antibodies on triplicate protein extracts of Nicotiana leaves transiently transformed with *35S::erYFP* or *35S::XVE-P2A-erYFP*. Arrow indicates a protein at ~27kDa, indicative of free erYFP protein derived from *XVE-P2A-erYFP*. Arrowhead indicates the predicted location at ~85kDa of a putative XVE-P2A-erYFP fusion protein. Each lane represents a sample from a separate leaf. N.I. is non-infiltrated leaf. **d-g** Confocal images of erCFP fluorescence in Nicotiana leaves, four days after co-infiltration of *p35S::XVE* and *pLexA::erCFP* (**d,f)** or *p35S;;XVE-P2A-erYFP* and *pLexA::erCFP* (**e,g**) constructs, in the presence (**d,e**) or absence (**f,g**) of 10 µM β-Est. β-Est and mock treatments in **d-g** were infiltrated 1 day prior to analysis. CFP fluorescence in cyan, transmitted light in grayscale. **h.** Western blot performed with anti-GFP antibodies on protein extracts of roots of pCO2B::erGFP, erYFP clone-bearing roots from TRAC-IT(pSCR>>mCherry/CFP) T3-11 and TRAC-IT(pWER>>mCherry/CFP) T3-18, and roots of wild-type Col-0. Arrow indicates a protein at ~27kDa, indicative of free erYFP protein derived from XVE-P2A-erYFP. Arrowhead indicates the predicted location at ~85kDa of a putative XVE-P2A-erYFP fusion protein. Scale bars: 50 µm

**Figure S2:** Marker lines showing the expression pattern of 6 experimentally validated tissue-specific promoters in the Arabidopsis root. The images shown in the right-hand panels of *pWER::erCFP, pCO2B::erGFP, pSCR::erYFP, pSHR::erGFP*, and pXPP::erGFP are recorded ~5mm from the root tip. Fluorescence of er-localised fluorescent proteins driven by the respective promoters is shown in false green, PI cell wall stain in magenta. Scale bars: 50 µm.

**Figure S3:** Intronization of *CRE-GR* prevents recombination of *TRAC-IT* plasmids in Agrobacterium. **a.** Representative image of constitutive erYFP fluorescence in a non-induced T1 seedling transformed with a first-iteration *TRAC-IT* construct, which lacks an intron in the *CRE-GR* gene driven by the *SCR* promoter. **b.** Agarose gel electrophoresis showing EcoRI digests of first-iteration *TRAC-IT* plasmid, isolated from *E. coli* and Agrobacterium. Agrobacterium samples were re-grown from seeds (lanes 1, 2) or from transformed colonies (lanes 3, 4). Arrows indicate fragments specific to non-recombined plasmid; arrowhead indicates the fragment specific to recombined plasmid. **c.** Agarose gel electrophoresis showing EcoRI digests of second-iteration *TRAC-IT* plasmid, identical to the construct in **b** but containing the intronized *CREi-GR* gene, isolated from *E. coli* and Agrobacterium (indicated as ‘Agro’) grown from transformed colonies. Arrows indicate fragments specific to non-recombined plasmid.

**Figure S4:** The *CREi-GR/LoxP-CRT1-LoxP* recombination system used in TRAC-IT generates cell-type-specific clones. **a.** Schematic of the simplified reporter-clone testing constructs used in these experiments. A tissue-specific promoter drives the CREi-GR recombinase, as in Module I of the TRAC-IT system. On the same construct, the constitutive pUBQ10 promoter drives the expression of a loxP-flanked norflurazon resistance gene (CRT1), which blocks transcription of the downstream erGFP reporter gene. DEX application activates the tissue-specific expressed CREi-GR that recombines the loxP sites thereby eliminating the CRT1 gene. erGFP now becomes expressed from the pUBQ10 promoter. **b.** Representative images from 12 independent T2 lines (2 lines for each promoter-construct) following a 24h induction on 10 µM DEX. GFP fluorescence in false green, PI cell wall stain in magenta. Differentiation zone (Diff. zone) sections were recorded ~1.5mm from the root tip except for pXPP::CREi-GR>>loxP-erGFP lines, which were imaged at ~10mm. Scale bar: 50 µm.

**Figure S5**: Increase in clone-derived YFP fluorescence in TRAC-IT roots following DEX application. **a-c**. Representative fluorescence binocular images from high-throughput screening of YFP fluorescence in roots of TRAC-IT(pWER>>mCherry/CFP) line T2-29 (**a**), TRAC-IT(pSCR>>mCherry/CFP) line T2-29 (**b**), and TRAC-IT(pXPP>>mCherry/CFP) line T2-13 (**c**) following a 24h treatment with 10 µM DEX (left panel) or a mock (right panel). Images in **c** are recorded ~1cm above the root tip. Seedlings were grown on nylon mesh to allow fast and easy transfer to clonal induction media, hence the “squared” autofluorescent background. Scale bars: 500 µm.

**Figure S6:** Relationship between T-DNA copy number (x-axis) and the percentage of root sections displaying non-induced clones (y-axis) across all 30 analysed TRAC-IT(pWER>>mCherry/CFP), TRAC-IT(pSCR>>mCherry/CFP) and TRAC-IT(pXPP>>mCherry/CFP) T2 lines. Fitted trendline is linear (R² = 0.66).

**Figure S7:** Tissue-specificity of clone formation following 24h induction with 10 µM DEX in the remaining 27 TRAC-IT lines. One representative confocal cross-section is shown for each line. Promoter pSCR, pWER or pXPP in combination with the particular T2 generation in each panel indicates the respective TRAC-IT line. Images for TRAC-IT(pXPP>>mCherry/CFP) lines were recorded ~1 cm above the root tip. YFP fluorescence in false green, Renaissance SR2200 cell wall staining in magenta. Scale bars: 50 µm.

**Figure S8:** Tissue-specificity of non-induced clone formation following 24h induction with a mock in the remaining 27 TRAC-IT lines. One representative confocal image is shown for each line. Promoter pSCR, pWER or pXPP in combination with the particular T2 generation in each panel indicates the respective TRAC-IT line. Images for TRAC-IT(pXPP>>mCherry/CFP) lines were recorded ~1 cm above the root tip. YFP fluorescence in false green, Renaissance SR2200 cell wall staining in magenta. Scale bars: 50 µm.

**Figure S9:** Representative confocal image showing autofluorescence in the CFP channel in the root meristem of wildtype Col-0 seedlings, recorded at the same settings as the root meristem images of TRAC-IT(pSCR>>mCherry/CFP) line T2-26 seedings in Figure 4. From left to right, panels show transmitted light, the CFP channel, and an overlayed image thereof. Scale bar: 50 µm.

**Figure S10:** Free mCherry signal in cells surrounding clones. **a.** Representative confocal image of YFP (left, green), mCherry (middle, magenta), and CFP (right, cyan) fluorescence in the transition zone of a TRAC-IT(pWER>>mCherry/CFP) T2-18 root that was incubated for 24h on 10 µM DEX followed by 24h on 10 µM β-Est. After imaging, the contrast setting of all channels was enhanced to highlight the presence of mCherry fluorescence in non-clone cells. A non-clone cell that clearly shows mCherry fluorescence is indicated with an arrow in the mCherry panel. **b.** Higher magnification confocal image of mCherry fluorescence (magenta) in non-clone cells, showing localisation to the cytoplasm and nucleus. Nuclear mCherry fluorescence indicated with arrows. **c.** Confocal image of mCherry fluorescence associated with a small, isolated clone in a TRAC-IT(pWER>>mCherry/CFP) T2-18 root that was incubated for 24h on a mock followed by 24h of 10 µM β-Est. The left panel shows YFP fluorescence in false green overlaid with transmitted light in grayscale. The right panel shows contrast-enhanced mCherry fluorescence, which gets fainter with increasing distance to the clone. mCherry fluorescence is shown with the Range Indicator scale (Zeiss), where red and blue respectively represent pixels with saturated and zero value and pixels in grayscale have intermediary values. Scale bars: **a** 50 µm; **b** 10 µm; **c** 25 µm.

**Figure S11:** Clone-derived regeneration from lateral roots of TRAC-IT(pWER>>PLT1/WOX5) line T3-23 seedlings. **a,b** Confocal images of *pWER::erCFP* expression in the epidermis of a stage VI lateral root primordium (**a**) and an emerged lateral root (**b**). CFP fluorescence is shown in false green, PI cell wall stain in magenta. **c,d** Presence of clones in the epidermis of a stage VI lateral root primordium (**c**) and an emerged lateral root (**d**) following a 24h induction with 10 µM DEX. YFP fluorescence is shown in false green, transmitted light in grayscale. **e,f** Representative confocal images of dividing clones in lateral roots after 24h induction with DEX followed by 3 (**e**) or 6 (**f**) days on 10 µM β-Est. YFP fluorescence in false green, WOX5-2xGFP in magenta, SR2200 cell wall staining in greyscale. Scale bars: 50 µm.

**Figure S12:** Representative images showing the reduced regeneration phenotype in mock-treated TRAC-IT(pWER>>PLT1/WOX5) line T3-23 seedlings. **a,b**. 6-day old seedlings were treated either with DEX for 24 h followed by mock for 21 days (**a**), or with mock for 24 h followed by β-Est for 21 days (**b**). The position of the root tip at the time of transfer to mock (**a**) or β-Est (**b**) is indicated with a black line. Arrow in **b** indicates an occasional regeneration event. Scalebar: 1cm.

**Figure S13:** Clone-specific accumulation of PLT1-3xmCherry and WOX5-2xGFP in TRAC-IT(pWER>>PLT1/WOX5) line T3-23. Representative confocal images, as elaboration on those used in Fig. 5 g-j, showing fluorescence of YFP, WOX5-2xGFP, and PLT1-3xmCherry in clones derived from the meristem (**a,b**) and differentiation zone (**c,d**) following subsequent induction of 24h on 10 µM DEX, and 3 (**a,c**) or 6 (**b,d**) days on 10 µM β-Est. Each confocal image is presented as four panels, showing from left to right: fluorescence of YFP (green) overlaid with SR2200 cell wall stain (grayscale), WOX5-2xGFP (blue), PLT1-3xmCherry (red), and an overlay of YFP, WOX5-2xGFP, and PLT1-3xmCherry in the same colour scheme. Scale bars: 50 µm.

**Figure S14:** Developing embryonic structures following induction of *PLT1* and *WOX5* originate from clones. **a,b** Representative confocal images of embryos regenerated form TRAC-IT(pWER>>PLT1/WOX5) line T3-23 seedlings that were sequentially treated with DEX for 24h followed by β-Est for 18 days. From left-to-right, panels show transmitted light in greyscale, SR2200 cell wall staining in magenta, YFP fluorescence indicative of clone identity in green, and an overlaid image thereof. Scalebar: 100µm

**Figure S15:** Epidermis-specific PLT1/WOX5-induced regeneration in TRAC-IT(pWER>>PLT1/WOX5) line T3-21. **a** Representative image of clone formation in the root meristem after 24h induction on DEX. YFP fluorescence in false green, PI cell wall stain in magenta. Arrow indicates an endodermal clone. **b** Representative image of clone formation in the root differentiation zone after 24h induction on DEX. YFP fluorescence in false green, transmitted light in greyscale. Arrow indicates a lateral root primordium. **c** Low-magnification image showing regeneration on roots that were sequentially treated with DEX for 24h followed by 10 µM β-Est for 22 days. The position of the root tip at the time of transfer to β-Est is indicated with a black line. **d, e** Higher-magnification images of regenerated shoots and embryos that formed on the part of the root that developed after transfer to β-Est (dashed boxes in **c**). **f-h** Representative confocal images of dividing epidermis-derived clones originating from the meristem (**f, g**) and the differentiation zone (**h**) after a 24h induction with DEX, followed by 3 (**f**) or 6 (**g, h**) days of induction on β-Est. Each confocal image is presented as four panels, showing from left to right: fluorescence of YFP (green) overlaid with SR2200 cell wall stain (grayscale), WOX5-2xGFP (blue), PLT1-3xmCherry (red), and an overlay of YFP, WOX5-2xGFP, and PLT1-3xmCherry in the same colour scheme. Scale bars: **a, b, f, g, h** 50 µm; **c** 1 cm; **d, e** 1 mm.
