# Supplementary material for: Tissue‐specific recombination‐activated clonal and inducible transcription (TRAC‐IT) enables controlled gene activation in stably marked tissues
Source: Plant J. 2026 Sep 28;127(6):e71151. doi: 10.1111/tpj.71151 (PMC13617364; doi:10.1111/tpj.71151)
Supplement: Supplementary file 3 [file TPJ-127-0-s004.docx]

**Table S1:** Performance of 30 independent T2 lines carrying *TRAC-IT(pWER>>mCherry/CFP)*, *TRAC-IT(pSCR>>mCherry/CFP), or TRAC-IT(pXPP>>mCherry/CFP)*, assessed by fluorescence microscopy. Line: (*) Clone formation also quantified by confocal microscopy; (†) Inducible fluorescent transgenes also studied by confocal microscopy. Copy number: number of T-DNA inserts determined by ddPCR. DEX-induced clones: (+) clearly increased clone YFP fluorescence after 24 h DEX treatment compared with mock. # roots with non-induced clones: at least 10 mock-treated roots per line were scored for clone fluorescence in the root meristem (pWER, pSCR) or a 5-mm differentiation zone section located 5–15 mm from the root tip (pXPP): (−) none, (+) 1–5 clones, (++) >5 clones. Region of clone formation: root region showing induced clones; distance measured from the tip of the root. Inducible fluorescent transgenes: (+) fluorescence detected only after β-estradiol induction; (−) no fluorescence detected; (*) constitutive fluorescence irrespective of β-estradiol treatment.

| Line | Copy number | DEX-induced clones | # roots with non-induced clones | | | Region of clone formation | Inducible fluorescent transgenes | |
| --- | --- | --- | --- | --- | --- | --- | --- | --- |
|  |  |  | **-** | **+** | **++** |  | *ermCherry* | *erCFP* |
| ***TRAC-IT(pWER>>mCherry/CFP)*** | | | | | | | | |
| T2-1*† | 1 | **+** | 7 | 2 | 1 | < 7 mm | **+** | **+** |
| T2-5 | 2 | **+** | 3 | 6 | 1 | < 9 mm | **+** | **+** |
| T2-11 | 2 | **+** | 9 | 1 | 0 | < 6 mm | **+** | **+** |
| T2-14 | 3 | **+** | 3 | 4 | 3 | < 7 mm | **+** | **+** |
| T2-17 | 2 | **+** | 3 | 7 | 0 | < 5 mm | **+** | **-** |
| T2-18† | 3 | **+** | 3 | 7 | 0 | < 9 mm | **+** | **+** |
| T2-23 | 2 | **+** | 4 | 6 | 0 | < 6 mm | **+** | **+** |
| T2-25† | 3 | **+** | 0 | 5 | 5 | < 8 mm | **+** | **+** |
| T2-29 | 2 | **+** | 6 | 4 | 0 | < 5 mm | **+** | **-** |
| T2-30 | 2 | **+** | 6 | 4 | 0 | < 5 mm | **+** | **+** |
| ***TRAC-IT(pSCR>>mCherry/CFP)*** | | | | | | | | |
| T2-6 | 4 | **+** | 3 | 6 | 1 | < 5 mm | **+** | **+** |
| T2-7 | 4 | **+** | 3 | 6 | 1 | < 5 mm | **+** | **+** |
| T2-8 | 3 | **+** | 5 | 5 | 0 | < 5 mm | **+** | **+** |
| T2-11† | 4 | **+** | 2 | 7 | 1 | < 5 mm | **+** | **+** |
| T2-14 | 4 | **+** | 1 | 8 | 1 | < 6 mm | **+** | **+** |
| T2-17† | 4 | **+** | 1 | 8 | 1 | < 6 mm | **+** | **+** |
| T2-18 | 3 | **+** | 5 | 5 | 0 | < 6 mm | **+** | **+** |
| T2-21 | 1 | **+** | 24 | 1 | 0 | < 2 mm | ***** | **-** |
| T2-26*† | 1 | **+** | 9 | 1 | 0 | < 5 mm | **+** | **+** |
| T2-31 | 2 | **+** | 8 | 2 | 0 | < 5 mm | **+** | **+** |
| ***TRAC-IT(pXPP>>mCherry/CFP)*** | | | | | | | | |
| T2-12 | 2 | **+** | 4 | 6 | 0 | > 3 mm | **+** | **+** |
| T2-13 | 3 | **+** | 2 | 4 | 4 | > 1.5 mm | **+** | **+** |
| T2-18 | 1 | **+** | 9 | 1 | 0 | > 2 mm | **-** | **-** |
| T2-20† | 4 | **+** | 0 | 2 | 8 | > 3 mm | **+** | **+** |
| T2-21 | 2 | **+** | 4 | 5 | 1 | > 3 mm | **+** | **-** |
| T2-24 | 1 | **+** | 14 | 1 | 0 | > 4 mm | **+** | **+** |
| T2-25 | 2 | **+** | 4 | 4 | 2 | > 3 mm | **+** | **+** |
| T2-29*† | 1 | **+** | 9 | 1 | 0 | > 4 mm | **+** | **+** |
| T2-31† | 2 | **+** | 7 | 3 | 0 | > 3 mm | **+** | **+** |
| T2-32 | 2 | **+** | 9 | 1 | 0 | > 5 mm | **+** | **+** |
